# ﻿Integrating morphological and genetic limits in the taxonomic delimitation of the Cuban taxa of Magnoliasubsect.Talauma (Magnoliaceae)

**DOI:** 10.3897/phytokeys.213.82627

**Published:** 2022-11-09

**Authors:** Ernesto Testé, Majela Hernández-Rodríguez, Emily Veltjen, Eldis R. Bécquer, Arlet Rodríguez-Meno, Alejandro Palmarola, Marie-Stephanie Samain, Luis R. González-Torres, Thierry Robert

**Affiliations:** 1 Jardín Botánico Nacional, Universidad de La Habana, Carretera “El Rocío” km 3½, 19230 Boyeros, La Habana, Cuba; 2 Ecologie Systématique et Evolution, Université Paris-Saclay, 360 Rue du Doyen André Guinier, 91405 Orsay, France; 3 Departamento de Biología Vegetal - Facultad de Biología, Universidad de La Habana, Calle 25 entre I y J, 10400 Vedado, La Habana, Cuba; 4 Ghent University Botanical Garden, Ghent University, K.L. Ledeganckstraat 35, 9000 Gent, Belgium; 5 Red de Diversidad Biológica del Occidente Mexicano, Instituto de Ecología, A.C., Avenida Lázaro Cárdenas 253, 61600 Pátzcuaro, Michoacán, México; 6 Systematic and Evolutionary Botany Lab, Department of Biology, Ghent University, K.L. Ledeganckstraat 35, 9000 Gent, Belgium; 7 Department of Biology, Douglas College, V3M 5Z5 New Westminster, British Columbia, Canada; 8 Département de Biologie, Sorbonne Université, 15-21 Rue de l’École de Médecine, 75006 Paris, France

**Keywords:** Gene flow, Genetic structure, integrative taxonomy, mclust, speciation, species delimitation

## Abstract

An accurate taxa delimitation, based on a full understanding of evolutionary processes involved in taxa differentiation, can be gained from a combination of ecological, morphological, and molecular approaches. The taxonomy of Magnoliasubsect.Talauma in Cuba has long been debated and exclusively based on traditional morphological study of a limited number of individuals. A more accurate description of leaf morphology variation using geometric morphometrics combined with genetic data could bring consistency to taxa delimitation in this group. Leaf samples for the morphological (243) and genetic (461) analyses were collected throughout the entire distribution range. The variability of each taxon was analyzed through multivariate and geometric morphometry, and 21 genetic markers (SSR). The observed leaf morphological variability was higher than previously described. Morphological and genetic classifications were highly congruent in two out of four taxa. Our data brought evidence that *Magnoliaorbiculata* can be considered a true species with very clear genetic and morphological limits. The main taxonomic issues concern the north-eastern Cuban populations of Magnoliasubsect.Talauma. The data supported the existence of two clear groups: corresponding mainly to *M.minor*-*M.oblongifolia* and *T.ophiticola*. However, these two groups cannot be considered fully delimited since genetic markers provided evidence of genetic admixture between them. Due to the likely absence of, at least strong, reproductive barriers between these three taxa, we propose therefore to consider them as a species complex.

## ﻿Introduction

Defining what a species is has been the subject of long debates in the history of biology, debates that have produced multiple species concepts (SC) over time (e.g. Genetic SC, Morphological SC, Phylogenetic SC, Ecological SC, Biological SC, among others (Mayr 1996; de Queiroz 2007). However, as pointed out by Hey (2006), this theoretical dilemma should not hinder the fact that biologists agree on simple and general ideas such as that species are fundamental units in biology, and that individuals belonging to the same species share a higher co-ancestry than with individuals from other species. The problem arises when one should define criteria for defining what a species is, and because criteria are linked to methodologies used to delineate species, therefore leading to different ways to define species (Hey 2006).

In the last two decades, there is an ever-growing shared idea that species can be defined as separately evolving metapopulation lineages (Unified SC) (de Queiroz 1998, 2007). Delimiting species boundaries, therefore, calls for accumulating evidence that the considered taxa are currently evolving independently. This task is especially challenging for taxa that have recently diverged, due to several evolutionary and genetic factors that have been described (see Naciri and Linder 2015, for a review). It is now largely recognized that an accurate taxa delimitation, based on a full understanding of evolutionary processes involved in taxon differentiation, can be gained from, and even should rely on, a combination of ecological, morphological, and molecular approaches to assess within-taxon diversity and among-taxa differentiation, across their whole geographical range (de Queiroz 2007; Padial et al. 2010).

The combination of several species concepts to broadly support species limits is known as integrative taxonomy (Padial et al. 2010). Dayrat (2005) and Will et al. (2005) recommended that species should only be named when their limits are supported by multiple lines of evidence. Integrative taxonomy does not replace traditional taxonomy but uses complementarity among disciplines to improve accuracy (Schlick-Steiner et al. 2010; Yeates et al. 2011). The potential for such integrative taxonomic approaches has not yet been fully embraced in botany, particularly in the tropics (Damasco et al. 2019). The works of Zheng et al. (2017), Alvarado-Sizzo et al. (2018), Damasco et al. (2019), Denham et al. (2019), Moein et al. (2019), Yang et al. (2019) are good examples of the use of an integrative approach carried out to solve the taxonomic problems in different plant families.

The genus *Magnolia* L. is a good model for applying an integrative taxonomic approach. It is the largest genus of the family *Magnoliaceae* Juss. It includes three subgenera, 13 sections, and an equal number of subsections (Figlar and Nooteboom 2004; Veltjen et al. 2022). The section Talauma Baill., with a Neotropical distribution, includes around 120 species distributed in four subsections: *Dugandiodendron* Lozano, *Chocotalauma* A. Vázquez, Á.J. Pérez and F. Arroyo, *Cubenses* Imkhan., and *Talauma* Juss. (Figlar and Nooteboom 2004; Vázquez-García et al. 2017). The 85 species of the subsection Talauma, the most species-rich of all *Magnolia* subsections, occur both in lowlands and mountainous areas (0 - 3 300 m.a.s.l.) of Central and South America, and the Caribbean Islands (Vázquez-García et al. 2017). The genus *Magnolia* includes species that could be perceived morphologically (Treseder 1978; Callaway 1994) and genetically (Lee and Chappell 2008; Li et al. 2013; Shen et al. 2018; Sun et al. 2020) conservative. Recent molecular research is challenging species delimitation based on morphology (Azuma et al. 2011; Rico and Gutierrez-Becerril 2019; Aldaba-Núñez et al. 2021). Most of these studies conclude that more evidence from ecology and morphology is needed, to understand the discrepancies with molecular data.

**Figure 1.**
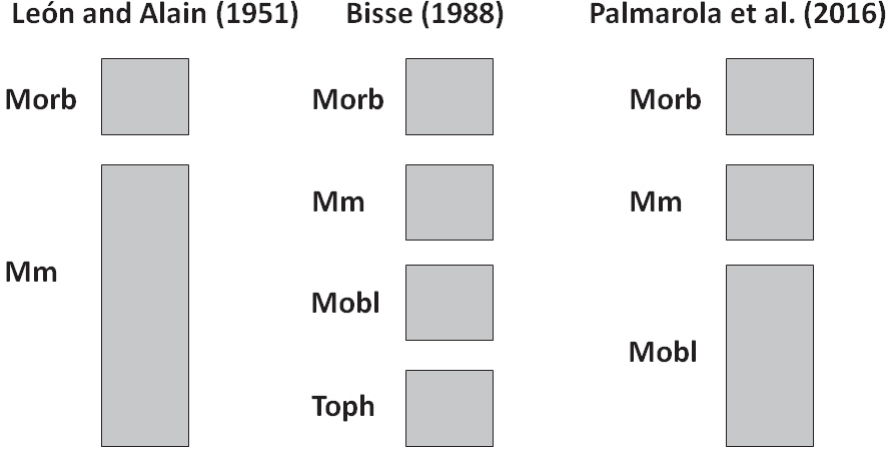
The three main classification systems (CS) of Magnoliasubsect.Talauma in Cuba. Morb: *M.orbiculata* (Britton & P. Wilson) Palmarola; Mm: *M.minor* (Urb.) Govaerts; Mobl: *M.oblongifolia* (León) Palmarola; Toph: *Talaumaophiticola* Bisse.

Cuba has the highest diversity of magnolias among the Caribbean islands, with seven endemic taxa (Veltjen et al. 2019). The Cuban taxa of *Magnolia* belong to two sections, *Magnolia* and *Talauma*. The section Talauma, the most diverse with six taxa, is represented by two subsections in Cuba: *Cubenses* and *Talauma*. The taxonomy of Magnoliasubsect.Talauma in Cuba has long been debated (Fig. 1, Suppl. material 7), although based until now only on leaf morphology. The first taxon described was *Talaumaminor* Urb. followed by *T.orbiculata* Britton and P. Wilson, in 1912 and 1923, respectively (Urban 1912; Britton 1923). Howard (1948) recognized two species of *Talauma*, *T.minor* Urb., and *T.truncata* (Moldenke) R.A. Howard, previously described by Moldenke (1946) as *Svenhediniatruncata* Moldenke. Two years later, León and Alain (1950) described a variety of *Talaumaminor* with extremely oblong leaves that they named T.minorvar.oblongifolia León. In the Flora of Cuba, León and Alain (1951) mentioned four taxa of *Talauma: T.minor* var. minor, T.minorvar.oblongifolia, *T.orbiculata* and *T.truncata*. These authors distinguished *T.orbiculata* and *T.truncata* based on the largely truncate leaves of the latter, which inhabits only areas around Pico Turquino (León and Alain 1951). However, due to the large variation of leaf-base shape observed in these two taxa, they were not recognized as separated entities in the subsequent taxonomic reviews of the group (e.g., Bisse 1988; Imkhanitzkaja 1993; Palmarola et al. 2016).

In the Supplemental Material of the Flora of Cuba, Alain (1969) suggested that all previously described taxa of *Talauma* from Cuba could be considered as one single taxon. Later, Borhidi and Muñiz (1971) considered *T.minor* as the only species of *Talauma* in Cuba and defined two subspecies: T.minorsubsp.oblongifolia (León) Borhidi and T.minorsubsp.orbiculata (Britton and P. Wilson) Borhidi. Afterward, Bisse (1974) described *T.ophiticola* Bisse and recognized *T.oblongifolia* (León) Bisse. Years later, Bisse (1988) referred four species: *T.orbiculata*, *T.minor*, *T.oblongifolia* and *T.ophiticola*. The delimitation of each taxon was mainly supported, as in previous works, by leaf morphological characters.

Based on anatomical and morphological (vegetative and reproductive) traits described by Nooteboom (1993), Frodin and Govaerts (1996) made the combination of *Talaumaminor* to *Magnoliaminor* (Urb.) Govaerts and considered all the other names of the Cuban *Talauma* species as synonyms of *M.minor* (Acevedo-Rodríguez and Strong 2012; Rivers et al. 2016). The latest taxonomic review of Magnoliasubsect.Talauma in Cuba recognized three species: *Magnoliaorbiculata* (Britton and P. Wilson) Palmarola, *Magnoliaminor*, and *Magnoliaoblongifolia* (León) Palmarola (Palmarola et al. 2016). In the absence of additional evidence, Palmarola et al. (2016) considered *T.ophiticola* synonym of *M.oblongifolia* due to the existence of one specimen (Bisse and Kohler HFC 5358 HAJB) that has leaves with the characteristics used by Bisse (1974, 1988) to define both taxa. A recent work (Testé et al. in press) analyzed the ecological niche of the group, concluding that *M.orbiculata* is the only species that could be considered ecologically distinct from the others.

All abovementioned taxonomic revisions (e.g., León and Alain 1950, 1951; Bisse 1974, 1988; Imkhanitzkaja 1993; Palmarola et al. 2016) were exclusively based on traditional leaf morphological descriptors and only a few individuals, limiting their ability to elucidate taxon boundaries. A more accurate description of leaf morphology variation using geometric morphometric combined with genetic data could significantly bring consistency to taxa delimitation in this group. The present work focuses on the Cuban taxa of Magnoliasect.Talaumasubsect.Talauma and aims to **(1)** assess their phenotypic variability of leaf morphological traits across their full geographic range **(2)** based on morphological data, evaluate the three main classification systems (called CS hereafter) of these taxa proposed up to date: the two taxa CS of León and Alain (1951), the four taxa CS of Bisse (1988), and the three taxa CS of Palmarola et al. (2016) (see Fig. 1); **(3)** infer the genetic structure of Magnoliasubsect.Talauma in Cuba; **(4)** integrate morphological and genetic data to review taxon delimitation in Magnoliasubsect.Talauma in Cuba.

## ﻿Materials and methods

### ﻿Sampling and taxon identification

**Figure 2.**
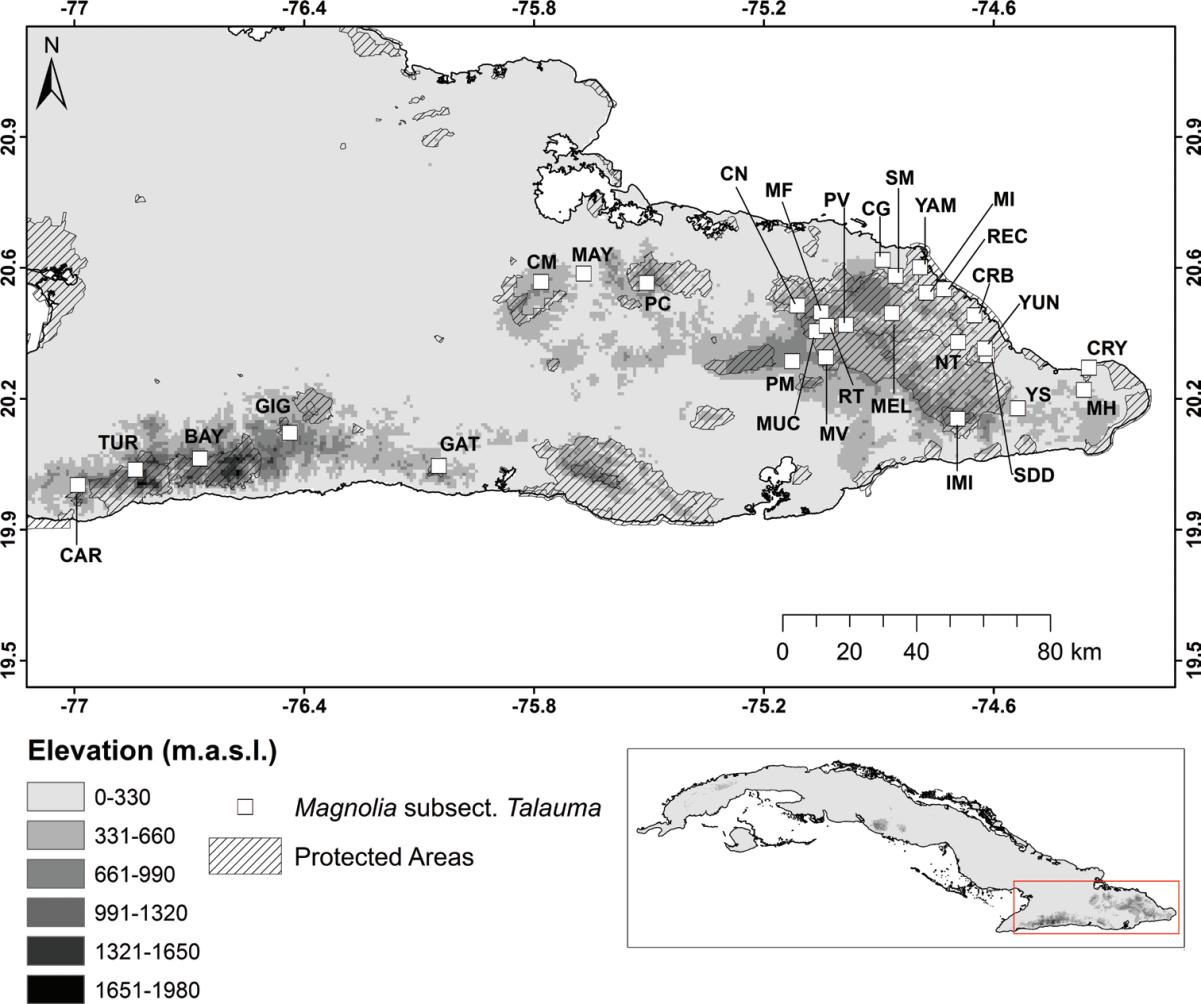
Geographic distribution of sampling locations of Magnoliasubsect.Talauma in Cuba.

The leaf samples for the morphological and genetic analyses were collected between 2015 and 2020 from individuals representing of Magnoliasect.Talaumasubsect.Talauma throughout their entire distribution range in the mountains of Nipe-Sagua-Baracoa and Sierra Maestra in eastern Cuba (Fig. 2). In the field, individuals were identified based on tree and leaf shape according to the morphological criteria outlined by Bisse (1988), because this author defined the highest number of species units (Fig. 1). León and Alain (1951) considered *T.truncata* an independent species. However, in the present work, individuals that could have been considered as *T.truncata*, were considered as part of the variability of *M.orbiculata*, as has been recognized by Bisse (1988), Imkhanitzkaja (1993), and Palmarola et al. (2016). To confirm species identity, 43 herbarium vouchers were collected or reviewed (Table 1). All herbarium vouchers were deposited in the Herbarium Johannes Bisse (HAJB, herbarium acronyms follow Thiers 2022) at the National Botanic Garden (University of Havana). The number of samples per species and localities is shown in Table 1.

**Table 1.** Recorded localities, demographic information, DNA samples and herbarium voucher of the taxa of Magnoliasubsect.Talauma in Cuba. HFC: “Serie Flora de Cuba”. All the samples were deposited in HAJB (Herbarium Johannes Bisse of National Botanic Garden-University of Havana). **NP**: National Park; **ER**: Ecological Reserve; **NOE**: Natural Outstanding Element; **PAMR**: Protected Area of Management resources. * Extinct in the locality; ? No field data available; NV: no voucher.

Taxa	Localities (AP)	Abrev.	Indiv.	Leaf	DNA	Voucher
*M.orbiculata*	NP Pico La Bayamesa	BAY	6	0	1	Molina Y. HFC 89590
*M.orbiculata*	NP Turquino	TUR	43	26	20	Palmarola A. & González-Torres L.R. HFC 89394
*M.orbiculata*	ER El Gigante	GIG	4	4	1	Testé E. HFC 90667
*M.orbiculata*	ER Pico Caracas	CAR	26	1	14	Palmarola A. et al. HFC 89194
*M.orbiculata*	Loma del Gato	GAT	?	4	0	León Hno. 23366
*M.minor*	Calizas de Mucaral (NP Alejandro de Humboldt)	MUC	35	9	16	Bécquer E.R. et al. HFC 90656
*M.minor*	Camarones-Río Báez (PAMR Cuchillas del Toa)	CRB	16	5	15	Bécquer E.R. et al. HFC 89579
*M.minor*	Cañón del Río Yumurí (NOE Cañón del Río Yumurí)	CRY	5	5	4	Bécquer E.R. et al. HFC 89829
*M.minor*	Cayo Guam	CG	43	3	22	Palmarola A. et al. HFC 89243
*M.minor*	Cayo Mujeres	CM	2	0	1	Palmarola A. et al. HFC 89213
*M.minor*	Cupeyal del Norte (NP Alejandro de Humboldt)	CN	34	4	19	Falcón B. et al. HFC 88955
*M.minor*	El Recreo (NP Alejandro de Humboldt)	REC	4	2	4	Bécquer E.R. et al. HFC 89467
*M.minor*	La Melba (NP Alejandro de Humboldt)	MEL	5	1	5	Palmarola A. et al. HFC 89584
*M.minor*	Mina la Hoya (NOE Cañón del Río Yumurí)	MH	29	9	12	NV
*M.minor*	Monte Fresco (NP Alejandro de Humboldt)	MF	18	0	12	García A. et al. HFC 90715
*M.minor*	Naranjo del Toa (NP Alejandro de Humboldt)	NT	15	7	13	NV
*M.minor*	Pico Cristal (NP Pico Cristal)	PC	16	13	15	Bécquer E.R. et al. HFC 89921
*M.minor*	Piedra La Vela (NP Alejandro de Humboldt)	PV	13	3	11	Bécquer E.R. et al. HFC 90519
*M.minor*	NOE Pinares de Montecristo	PM	33	8	16	Bécquer E.R. et al. HFC 90421
*M.minor*	Región del Toa (NP Alejandro de Humboldt)	RT	29	7	15	Bécquer E.R. et al. HFC 90660
*M.minor*	Río Yamanigüey (NP Alejandro de Humboldt)	YAM	72	5	28	Bécquer E.R. et al. HFC 89449
*M.minor*	Sur de las Delicias del Duaba	SDD	2	1	2	Díaz J. et al. HFC 89435
*M.minor*	Yumurí del Sur	YS	8	5	5	Bécquer E.R. et al. HFC 89510
*M.minor*	NOE Yunque de Baracoa	YUN	3	2	3	Bisse J. HFC 5321
*M.minor*	Siera de Imías	IMI	?	2	0	Alvarez A. et al. HFC 27534
*M.minor*	Presa de Cola de Moa	–	*	1	0	Wright 1100
*M.minor*	Presa de Mayarí	–	*	1	0	Shafer 8335
*M.oblongifolia*	Calizas de Mucaral (NP Alejandro de Humboldt)	MUC	1	1	1	Bécquer E.R. et al. HFC 90655
*M.oblongifolia*	Cayo Guam	CG	11	11	3	Palmarola A. et al. HFC 89249
*M.oblongifolia*	Cupeyal del Norte (NP Alejandro de Humboldt)	CN	31	12	15	Falcón B. et al. HFC 88959
*M.oblongifolia*	La Melba (NP Alejandro de Humboldt)	MEL	5	0	2	Palmarola A. et al. HFC 89589
*M.oblongifolia*	Pico Cristal (NP Pico Cristal)	PC	5	4	3	Bécquer E.R. et al. HFC 89933
*M.oblongifolia*	Piedra La Vela (NP Alejandro de Humboldt)	PV	4	3	3	Bécquer E.R. et al. HFC 90543
*M.oblongifolia*	Río Yamanigüey (NP Alejandro de Humboldt)	YAM	6	6	6	Bécquer E.R. et al. HFC 89452
*M.oblongifolia*	Sur de las Delicias del Duaba	SDD	1	1	1	Díaz J. et al. HFC 89435
*M.oblongifolia*	Yunque de Baracoa	YUN	2	2	1	Bécquer E.R. et al. HFC 89531
*T.ophiticola*	Cayo Guam	CG	130	15	33	Bécquer E.R & Testé E. HFC 89439
*T.ophiticola*	Cupeyal del Norte (NP Alejandro de Humboldt)	CN	82	23	39	Falcón B. et al. HFC 88950
*T.ophiticola*	La Melba (NP Alejandro de Humboldt)	MEL	12	5	12	Palmarola A. et al. HFC 89587
*T.ophiticola*	Mina Iberia (NP Alejandro de Humboldt)	MI	77	16	45	Palmarola A. et al. HFC 89261
*T.ophiticola*	Monte Fresco (NP Alejandro de Humboldt)	MF	11	0	8	NV
*T.ophiticola*	Pico Cristal (NP Pico Cristal)	PC	8	6	7	Bécquer E.R. et al. HFC 89917
*T.ophiticola*	Piedra La Vela (NP Alejandro de Humboldt)	PV	4	0	3	Bécquer E.R. et al. HFC 90531
*T.ophiticola*	Subida a la Melba (km 10)	SM	7	0	7	Alvarez A. et al. HFC 42531
*T.ophiticola*	Sur de las Delicias del Duaba	SDD	12	8	10	Bécquer E.R. et al. HFC 89556
*T.ophiticola*	NOE Yunque de Baracoa	YUN	19	2	8	Bécquer E.R. et al. HFC 89529

For the morphological analyses, 4–8 healthy leaves from 200 individuals were randomly collected, across the entire range of taxa within each locality. A leaf was considered healthy if the full outline of the leaf was undamaged. Leaves were photographed with a Nikon camera on a white background with a fixed ruler. The petiole of the leaf was removed before taking pictures, and the camera was mounted on a tripod to standardize the angle and distance of the photographs. To expand the geographic scope of our study, we also included leaf samples from 43 herbarium specimens (deposited in HAC, HAJB, and B). Hence, in total 243 individuals of Magnoliasect.Talaumasubsect.Talauma in Cuba were morphologically analyzed.

For the genetic analyses, young leaf samples of a total of 461 individuals, belonging to 26 of 30 known localities, were stored in self-sealed bags with silica gel for DNA extraction. The resulting number of DNA samples represented 52% of the known individuals of Magnoliasubsect.Talauma in Cuba (close to 900 individuals).

### ﻿Multivariate and geometric morphometry

Analyses based on morphological variables were aimed at comparing the relevance of each of the three CS previously proposed: the two taxa CS, *Magnoliaminor* and *M.orbiculata*, of León and Alain (1951); the four taxa CS, *M.minor*, *M.orbiculata*, *M.oblongifolia*, and *Talaumaophiticola*, of Bisse (1988); and the most recent, the three taxa CS, *M.minor*, *M.orbiculata*, and *M.oblongifolia*, of Palmarola et al. (2016) (Fig. 1). Two types of morphological analyses were carried out on three independent datasets: 1) multivariate morphometry analysis: a linear and angular measures dataset, 2) geometric morphometry analysis: an outline dataset and a landmarks coordinates dataset.

**Figure 3.**
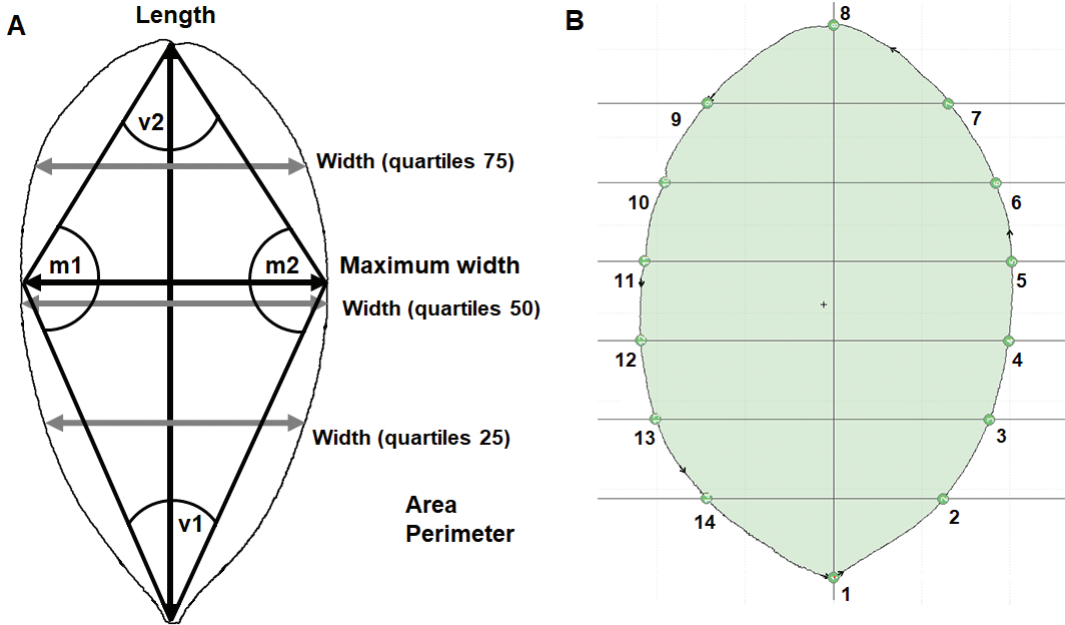
**A** the 11 morphological variables measured on leaves of Magnoliasubsect.Talauma in Cuba; v1 = angle of the base, v2 = angle of the apex; m1 and m2 = lateral angles in the maximum wide **B** quadratic grid with six lines and the position of the 14 landmarks (type 1: points 1 and 8; type 2: the other 12 points), placed on the leaves of Magnoliasubsect.Talauma in Cuba.

In the multivariate morphometry analysis, linear and angular measures of leaf characters were automatically taken from the digital photographs using the R v. 3.4.1 (R Core Team 2017) package FOLIOMETRIK v. 0.2.2 (Ramírez-Arrieta and Denis 2020). Eleven leaf variables were measured: central axis length (Length), maximum width, width at the three main quartiles (25, 50, and 75 quartiles), the perimeter of the contour (Perimeter), surface area (Area), and internal angles (v1 = angle of the base, v2 = angle of the apex; m1 and m2 = lateral angles at the maximum width) (Fig. 3A). Additional to the eleven measured variables, we calculated the maximum width/length ratio, named Calculated Index of Bisse (B_ci_) for each leaf. The eleven variables were recorded for each leaf. Subsequently, the twelve variables were averaged per individual for the 4–8 leaves available per individual. These averages of the twelve variables were used for all the subsequent statistical analyses.

In the geometric morphometry analysis, the outline dataset was obtained through a semi-automated shape analysis performed in FOLIOMETRIK v. 0.2.2 (Ramírez-Arrieta and Denis 2020). We set the program outputs to the Elliptic Fourier Descriptors (EFDs) (Jensen 2003), to obtain the first 25 harmonics (Chuanromanee et al. 2019). The harmonics were normalized to eliminate the differences in size, position, rotation, and starting point. This allowed removing the undesired experimental source of random variation and analyzing true differences of leaf shape between individual measurements (Jensen 2003). The landmarks coordinates dataset was obtained as follows. The positions of landmarks were determined by placing a quadratic grid with six lines on each leaf. In between the intersections of the grid and the border of the leaf we set 14 landmarks, two of them anatomical (type 1, i.e. apex and base) and the other 12 mathematically defined (type 2) (Fig. 3B). All analyses were carried out in FOLIOMETRIK v. 0.2.2. The landmarks X and Y coordinates were standardized using a Generalized Procrustes Analysis in PAST v. 2.14 (Hammer et al. 2001). Next, two variables: the Sum EDMA (Euclidean Distance Matrix Analysis) and centroid size were calculated.

The statistical significance of the differences among taxa for each measured variable (linear and angular variables, Sum EDMA, and centroid size) was assessed by a MonteCarlo analysis in PopTools v. 3.23 (Hood 2010) with 10 000 random permutations. The variability of the whole sample was described by using a normalized Principal Component Analysis (PCA). Differences among taxa were tested according to a one-way nonparametric MANOVA, using Euclidian distance, with 10 000 randomizations. Correction of p-values for multiple testing was done using the Bonferroni method. The multivariate comparisons were done independently for each dataset (linear and angular measures dataset, outline datasets, and landmarks coordinate dataset). All statistical analyses were conducted in R v. 3.4.1 (R Core Team 2017) and PAST v. 2.14 (Hammer et al. 2001), and the threshold used to decide for statistical significance was a p-value of 0.001.

### ﻿Clustering analysis based on morphological variability

A Bayesian clustering approach based on Gaussian finite mixture models was carried out using each of the three datasets of morphological variables using the “mclust” R package (Scrucca et al. 2016). The method tests the number of clusters and different mixture models that best fit the data according to the number of clusters (G) chosen a priori. The method allows comparing the quality of the discrimination among clusters based on the Bayesian Information Criteria (BIC) allowing to choose the best value(s) of G, without any information about individual assignation to the different clusters. The default “mclust” setting was used to assess the 14 types of models which all differ in the covariance matrix landscape (see Scrucca et al. 2016; Zhang and Di 2020, for further details about the models). We varied G values between 1 and 9 (default option). Three independent analyses were performed using the three datasets; to compare the power of those three groups of variables to discriminate among taxa. Because the analyses sometimes provided clusters with only one individual, those clusters were considered “ghost” clusters and not considered as true clusters.

### ﻿DNA extraction and PCR

DNA was extracted from dried leaf tissue using a modified cetyltrimethylammonium bromide (CTAB) extraction protocol (Doyle and Doyle 1990) with MagAttract Suspension G solution mediated cleaning (Xin and Chen 2012). DNA quality was assessed using a spectrophotometer NanoDrop 1000 Spectrophotometer (Thermo Fisher Scientific, Waltham, MA, USA). Individuals were genotyped using 21 microsatellite markers (simple sequence repeats, SSR) (Suppl. material 8) developed on four Neotropical *Magnolia* species: *M.lacandonica* A. Vázquez, Pérez-Farr. and Mart.-Camilo (MA39), *M.mayae* Vázquez and Pérez-Farrera (MA40), *M.dealbata* Zucc. (MA41) and M.cubensissubsp.acunae Imkhan. (MA42) (Veltjen et al. 2019), using four-primer PCR multiplex method (Vartia et al. 2014). PCR conditions and primer labeling followed Veltjen et al. (2019). The combination and parameters of the four multiplex reactions are given in Suppl. material 8. The lengths of the DNA fragments were detected using an ABI 3130XL fragment analyzer, quantified with a GeneScanTM 500 LIZ size standard (Thermo Fisher Scientific), and analyzed in Geneious v. 8.0.5 (Kearse et al. 2012) with the microsatellite plugin.

### ﻿Genetic structure

Genetic diversity values were calculated for each taxa using GeneAlex v. 6.5 (Peakall and Smouse 2012) and Genepop v. 4.7.5 (Rousset 2008). Genetic differentiation between taxa was estimated through pairwise comparisons of F_ST_ (Weir and Cockerham 1984) and D_JOST_ values (Jost 2008) using the fastDivPart function of the R package diveRsity (Keenan et al. 2013). The identification of genetic clusters and the assignment of individuals was performed using STRUCTURE v. 2.3.4 (Pritchard et al. 2000), which uses a Bayesian clustering approach using MCMC for posterior distribution sampling. STRUCTURE analyses were conducted using a model that assumes admixture, correlated allele frequencies, and without prior population information. First, 10 replicates were run for each genetic clusters (K), with K varying between 1 to 20 and a burn-in period of 50 000 iterations followed by a run-length of 150 000 iterations of the Markov Chain. The most probable number of groups was determined according to the method of Evanno et al. (2005) as implemented in STRUCTURE HARVESTER v. 0.6.94 (http://taylor0.biology.ucla.edu/structureHarvester) (Earl and von Holdt 2012). Then, 100 new repetitions of the MCMC method were run for the best K value. CLUMPP v. 1.1.2 (Jakobsson and Rosenberg 2007) was used to estimate similarities between runs and to average the membership probabilities. Final bar plots displaying individual admixture coefficients were obtained thanks to Structure Plot v. 2.0 (Ramasamy et al. 2014). An individual was considered a member of a genetic group when its probability of belonging to that group was higher than or equal to 0.9. A second STRUCTURE analysis was executed (using the same configuration) without considering the individuals of *M.oblongifolia* (sensu Bisse 1988).

Because the MCMC method implemented in STRUCTURE is based on a population genetic model, the results of genetic clusters and assignment of individuals, may be affected by the potential low model fit to data. Thus, a non-model-based multivariate clustering analysis was also performed. A DAPC analysis (Discriminant Analysis of Principal Components) was executed in R v. 3.6.1 (R Core Team 2017) using the adegenet R package (Jombart et al. 2010). Firstly, a PCA was run on the whole dataset for which the first 200 Principal Components (PCs) were retained. Secondly, a discriminant analysis was executed using the number of genetic clusters defined in the previous step. Parallel to the STRUCTURE analysis, a second DAPC analysis was done without *M.oblongifolia* (sensu Bisse 1988). An individual was considered a member of a genetic group when its probability of belonging to that group was higher than or equal to 0.9.

For all analyses, graphical representations of outputs were built using the four taxa CS to have a representative overview of the correspondence between genetic clusters and each already defined taxon.

### ﻿Integrating morphological and genetic limits

Because 138 individuals were analyzed both at the morphological and the genetic level, the correspondence between the groups inferred from both type of characters was assessed. The distributions of individual assignment to each morphological (mclust) and genetic (STRUCTURE) clusters were compared with a Chi^2^ test carried out on the Contingency Assignment Table using PAST v. 2.14 (Hammer et al. 2001). A heatmap was made, in R v. 3.4.1, to analyze the variation in the cluster assignation inside each taxon.

## ﻿Results

### ﻿Multivariate and geometric morphometry

**Figure 4.**
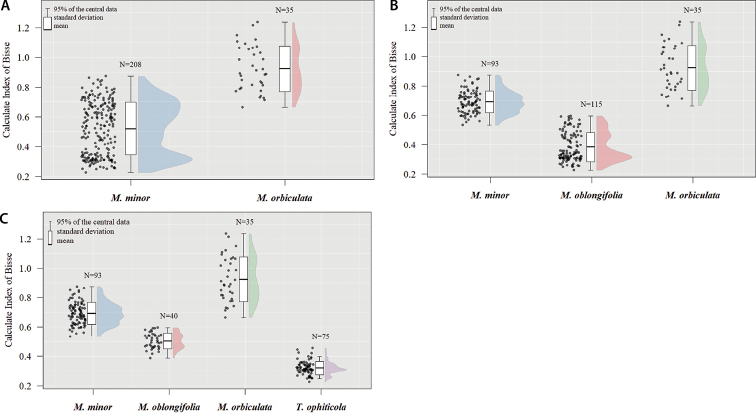
Graphic representation of Calculated Index of Bisse (B_CI_) measured in the individuals of Magnoliasubsect.Talauma in Cuba. Two taxa CS (**A**), four taxa CS (**B**), three taxa CS (**C**).

**Figure 5.**
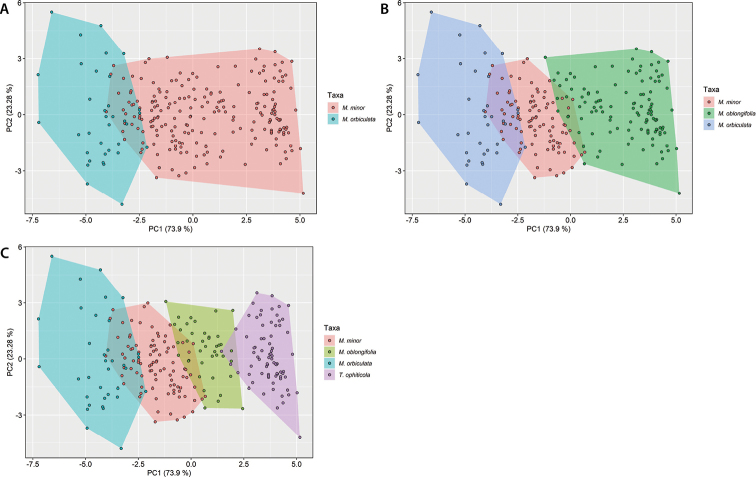
Principal Component Analysis for the multivariate morphometric variables measured in the individuals of Magnoliasubsect.Talauma in Cuba. Two taxa CS (**A**), three taxa CS (**B**), four taxa CS (**C**).

The results of the multivariate morphometry analysis are summarized in Figs 4, 5, Suppl. materials 1–3, 9. The Calculated Index of Bisse (B_ci_) showed significant differences between the defined species whatever the CS tested (p < 0.0001). *Magnoliaorbiculata* and *Talaumaophiticola* displayed the highest and the lowest mean B_ci_, respectively. Most of the eleven other variables showed significant differences between taxa, whatever the CS (Suppl. materials 1–3). There were three exceptions. The leaf perimeter did not show significant differences between *Magnoliaminor* and *Talaumaophiticola* (p = 0.211), and between *M.oblongifolia* and *T.ophiticola* (p = 0.132) when the four taxa CS was considered. Likewise, the leaf area between *M.minor* and *M.oblongifolia* (p = 0.115) did not show differences when the three taxa CS was used. When following the two taxa CS (Fig. 4A, Suppl. material 1), eight variables (Maximum width, B_ci_, Width-quartiles 50, Width-quartiles 75, Internal angles-v1, Internal angles-m1, Internal angles-v2, Internal angles-m1) showed an intra-taxon bimodal pattern within *M.minor*.

**Figure 6.**
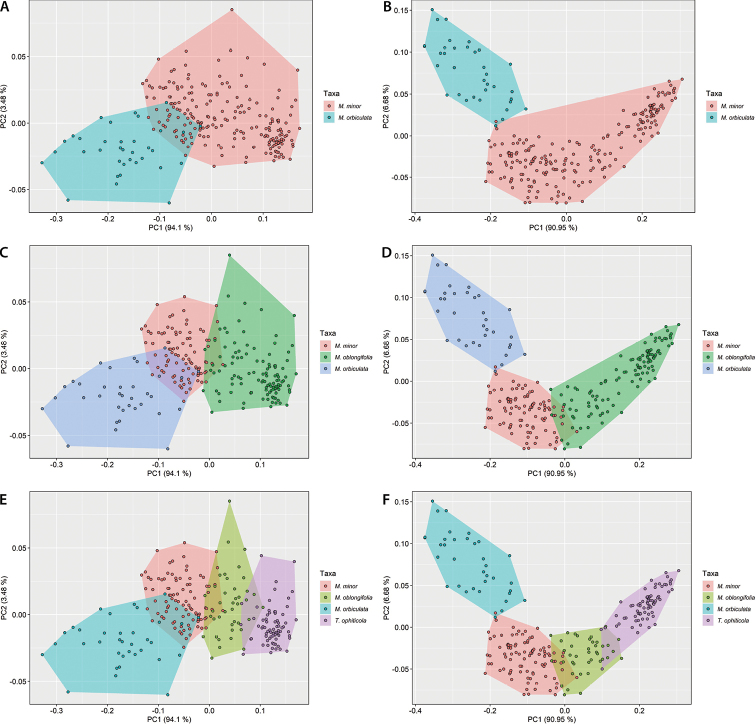
Principal Component Analysis for the Elliptic Fourier Descriptors (**A, C, E**) and Coordinates of the landmark (**B, D, F**) which characterized the leaves of Magnoliasubsect.Talauma in Cuba. Two taxa CS (**A, B**), three taxa CS (**C, B**), four taxa CS (**E, F**).

Despite the clear morphological differentiation between taxa, overlap in the multivariate distributions of leaf morphology variables was observed (Fig. 5). The internal angle of the base (-0.324) and the leaf perimeter (0.5627) displayed the highest weight in the first two principal components, respectively (Suppl. material 9). The NPMANOVA showed significant statistical differences (p < 0.0001) between taxa for each of the CS (Suppl. material 10). The comparison between groups, based on Sum EDMA and centroid size, showed significant differences for most comparisons (Suppl. material 4). The exceptions were: the Sum EDMA between *M.minor* and *M.oblongifolia* (p = 0.316) and between *M.orbiculata* and *T.ophiticola* (p = 0.406), when referring to the four taxa CS (Suppl. material 4). Fig. 6 illustrates PCAs on the outline dataset (Fig. 6A, C, E), and the landmark dataset (Fig. 6B, D, F) for the two (Fig. 6A, B), three (Fig. 6C, D) and four (Fig. 6E, F) taxa CS. Based on PCA for elliptic Fourier descriptors and Landmark, the different taxa had little overlap in the ordination space (Fig. 6). However, a clearer distinction among taxa was obtained with landmark positions than with other quantitative variables. This was especially obvious with *M.orbiculata*, which was strongly differentiated from other taxa when using landmark positions, no matter the CS considered.

The NPMANOVA showed significant statistical differences (p < 0.001) between taxa for each of the CS in the linear and angular measures dataset (Suppl. material 10). Similarly, the NPMANOVA showed significant statistical differences (p < 0.001) between the groups in the outline and landmark datasets (Suppl. material 10).

### ﻿Clustering analysis based on morphological variability

**Figure 7.**
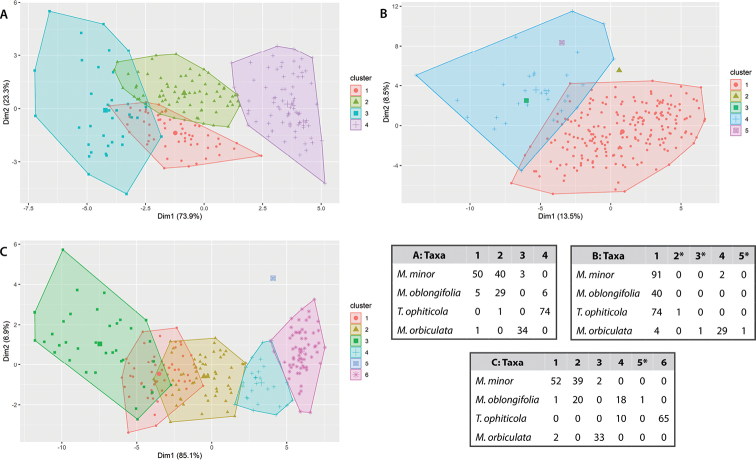
Graphic representation and classification matrix obtained after the cluster analysis using the morphological data of Magnoliasubsect.Talauma in Cuba. **A** Linear and angular variables **B** elliptic Fourier Descriptors **C** matrix of landmarks. * Ghost Cluster.

The clustering analysis based on morphological variability showed differences in the number of groups inferred by the best models, according to the different datasets (Fig. 7; Suppl. material 11). The highest BIC scores were retrieved for G = 4 for linear and angular dataset, G = 2 for the Elliptic Fourier Descriptors dataset (with other 3 ghost clusters), and G = 6 for the Landmark dataset (with other ghost clusters) (Fig. 7; Suppl. material 11). It was noticeable that for each data set, the probabilities of assignment of each individual were higher than 0.9 in all cases based on the Elliptic Fourier Descriptors. In the case of the linear and angular variables and matrix of landmarks, only 5 and 22 individuals showed probabilities of an assignment less than 0.9, respectively (data not shown). The linear and angular variables allowed a clear discrimination between *M.orbiculata*, *T.ophiticola* and *M.minor*, the latter taxa being split into two clusters. One of these two clusters was shared only with the majority of *M.oblongifolia* individuals.

The clustering analysis based on Elliptic Fourier Descriptors provided only two clusters (Fig. 7). The assignment of individuals was therefore different from that obtained with linear and angular variables. Indeed, all individuals of *M.oblongifolia*, and most individuals of *T.ophiticola* and *M.minor*, were assigned to the same cluster (cluster 1), while most individuals of *M.orbiculata* were assigned to a different cluster (cluster 4). Therefore, Elliptic Fourier descriptors were efficient to discriminate between *M.orbiculata* on the one hand and the 3 other taxa on the other hand. Finally, the analysis carried out on the matrix of landmarks showed a similar pattern to that obtained with the linear and angular variables for *M.minor*, *T.ophiticola* and *M.orbiculata*. The main difference between these two analyses (matrix of landmarks and linear and angular dataset) was that in the first one, *M.oblongifolia* was split into two clusters, one of which was shared with *M.minor* and the other one with *T.ophiticola* (Fig. 7).

Thus, despite a continuous variation of leaf morphology across taxa, a clear delimitation of *M.orbiculata* is shown by our analyses whichever data set was used. In cases where individuals of the same taxon were assigned to different clusters, no obvious correspondence between the assigned clusters and the geographic origin of those individuals was found. Indeed, many individuals of the same taxon/locality were assigned to different clusters (data not shown).

### ﻿Genetic structure and taxon differentiation

**Figure 8.**
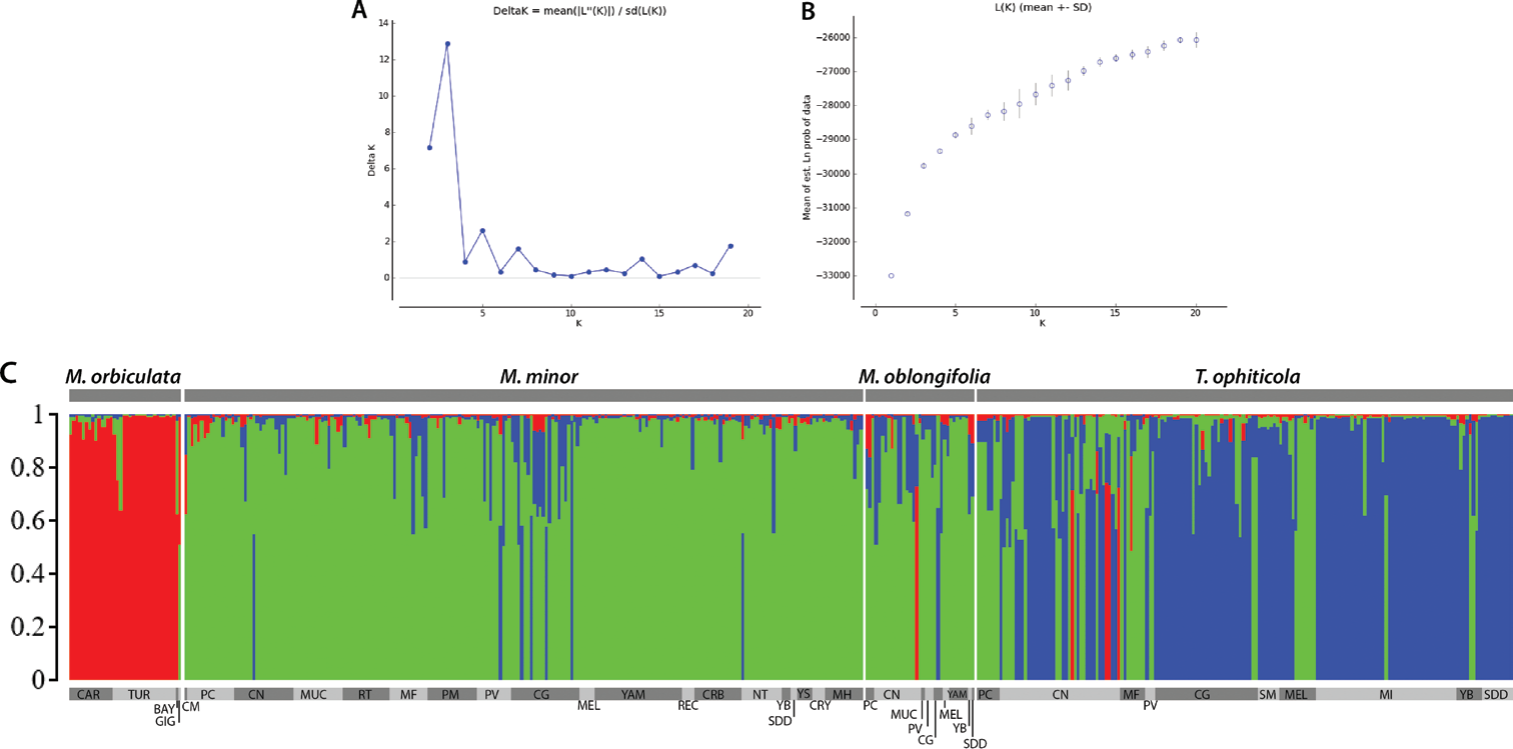
Structure results of Magnoliasubsect.Talauma in Cuba for the complete dataset **A** Delta K plot **B** the mean Ln(K) plot **C** representative bar plot (out of 100 en replicates) for K = 3.

The species with the greatest genetic diversity were *Magnoliaminor* and *Talaumaophiticola*, while the lowest diversity was found in *Magnoliaorbiculata*. The expected heterozygosity was similar in the four taxa (Table 2). The genetic differentiation among taxa was relatively high (global F_ST_ = 0.10, D_JOST_ = 0.23). *Magnoliaorbiculata* contributed mainly to this result since it was highly differentiated from the three other taxa, while *M.minor* and *M.oblongifolia* were the less differentiated taxa (Table 3). The Bayesian clustering analysis clearly provided three genetic clusters as the unambiguously best solution in the two analyses (with and without *M.oblongifolia*) (Fig. 8A, B, Suppl. material 5: fig. S5A, B). In the following, an individual was considered to be correctly assigned to a unique genetic cluster if the ancestry coefficient of this individual to this cluster was higher than or equal to 0.9. One of those clusters corresponded obviously to *M.orbiculata* (red cluster in Fig. 8C). The 88.8% (32/36) of individuals from *M.orbiculata* were assigned to this cluster, while the 4/36 *M.orbiculata* individuals were considered unclear. The second cluster (green cluster in Fig. 8C) consisted mainly of the majority of *M.minor* (171) *and M.oblongifolia* (16) individuals, but also included some individuals (14) of *T.ophiticola*. (Fig. 8C). The third cluster (blue cluster on Fig. 8) was predominantly composed of *T.ophiticola* with only one individual of *M.minor*. We will therefore refer hereafter to the “orbiculata”, “minor-oblongifolia” and “ophiticola” genetic clusters, keeping in mind that ancestry coefficients within each taxon of these genetic clusters still varied. Indeed, despite a clear delimitation between three genetic clusters, a significant proportion of individuals (130/461) displayed genetic admixture (on the basis of a 0.9 admixture coefficient value as a threshold). Based on these “admixed” individuals, the level of genetic admixture varied according to taxa. The mean value of probability to belong to their a priori cluster (defined from their taxonomic status) was 0.624 (±0.104) for *M.orbiculata*, 0.678 (±0.172) for *M.minor* (including *M.oblongifolia*), and only 0.477 (±0.262) for *T.ophiticola*.

**Table 2.** Average values and standard deviation of the measures of genetic diversity by taxa of Magnoliasubsect.Talauma in Cuba. N: sample size, N_P_: number of private alleles, N**_A_**: number of mean alleles, A_R_: allele richness, N_E_: number of effective alleles, He: expected heterozygosity.

Taxa	N	Np	Na	Ar	Ne	He
***M.orbiculata***	36	0.524±1.030	6.81±1.18	6.652±5.237	3.618±0.543	0.564±0.064
***M.minor***	218	2.286±2.217	13.619±2.043	9.091±6.472	5.732±1.169	0.588±0.072
***M.oblongifolia***	35	0.333±0.483	9.810±1.360	9.674±6.134	5.420±0.929	0.630±0.065
***T.ophiticola***	172	1.619±2.037	12.524±1.896	9.163±5.986	5.658±1.023	0.650±0.057

**Table 3.** Pairwise genetic differentiation measures: fixation indices (F_ST_) (below diagonal) and allelic differentiation index (D_JOST_) (above diagonal) calculated for the taxa of Magnoliasubsect.Talauma in Cuba. In all case significant differences were found (p ˂ 0.001).

Taxa	*M.orbiculata*	*M.minor*	*M.oblongifolia*	*T.ophiticola*
*M.orbiculata*	–	0.3127	0.2937	0.3921
*M.minor*	0.1721	–	0.0056	0.0999
*M.oblongifolia*	0.1613	0.0092	–	0.0705
*T.ophiticola*	0.1982	0.0859	0.045	–

*Magnoliaorbiculata* was strongly homogeneous pertaining to ancestry coefficient values with only four individuals displaying genome admixture with the “minor-oblongifolia” cluster (Fig. 8C). This is strongly in agreement with what was observed for leaf characteristics. *Magnoliaminor* and *M.oblongifolia* displayed a high level of genome admixture with the “ophiticola” cluster. 35 individuals (16.1%) of the individuals of *M.minor* showed genome admixture with the “ophiticola” cluster. In *M.oblongifolia*, 51% of the individuals exhibited an ancestry coefficient over 0.9 to the “minor-oblongifolia” cluster, the rest showed high admixture levels. Moreover, it is noticeable that one individual of *M.oblongifolia* displayed a very high ancestry to *M.orbiculata*. The localities of Cupeyal del Norte (CN), Monte Fresco (MF), Piedra la Vela (PV), and Cayo Guam (CG) show the highest levels of misclassification of *M.minor* and *M.oblongifolia* into the “ophiticola” cluster.

For *T.ophiticola*, 56.4% (97/172) of individuals could be assigned to the “ophiticola” genetic cluster while 8.14% (14/172) could be assigned to the “minor-oblongifolia” genetic cluster (referred to as “misclassified” individuals hereafter). Similar to *M.minor* and *M.oblongifolia*, many individuals of *T.ophiticola* (61/172) also displayed signals of genetic admixture, mainly with the “minor-oblongifolia” cluster, but also, for a few of them, with the “orbiculata” cluster. The localities of Subida a la Melba (SM), Mina Iberia (MI), and Sur de las Delicias del Duaba (SDD) showed the lowest levels of misclassification. Four individuals from Cupeyal del Norte (CN) were clustered with the group of *M.orbiculata.* Most individuals from La Melba (MEL), Pico Cristal (PC), and Monte Fresco (MF) showed an ancestry coefficient similar to the “minor-oblongifolia cluster”. The clustering analysis without individuals of *M.oblongifolia* also provided K = 3 as the best solution (Suppl. material 5: fig. S5A, B). Moreover, it was striking that this analysis provided an ancestry pattern very similar (Suppl. material 5: fig. S5C) to the analysis including this taxon (Fig. 8C). This demonstrated the very good stability of inferences on individuals’ ancestry coefficients which could be explained by the strong genetic delimitation between the three identified genetic clusters.

The PCA analysis on the whole SSR data set showed that the 200 first principal components explained 99.3% of the variation, which were therefore kept for the discriminant analyses. Based on the number of taxa that have been defined across the history of Cuban *Talauma* taxonomy, but also on the STRUCTURE results, two solutions for the number of genetic clusters were considered in the following discriminant analysis (DAPC) K = 3 and K = 4. When K = 3, individual assignment displayed a pattern very similar to that found with the Bayesian clustering approach; with one cluster predominantly composed by *M.minor* and *M.oblongifolia*, the other cluster with *T.ophiticola*, and the third one with the individuals of *M.orbiculata*. In the three clusters, some level of misclassification was found. Many individuals “misclassified” in the DAPC analysis were the same that were “misclassified” based on the STRUCTURE analysis. The DAPC analysis confirmed the correspondence of *M.orbiculata* to a unique genetic cluster as expected because of its high genetic differentiation from the three other taxa (Suppl. material 6: fig. S6A). For K = 3 only one individual of *T.ophiticola* showed an assignment probability value less than 0.9.

K = 4 (Suppl. material 6: fig. S6B), seems to be a less meaningful solution. In this case, three clusters were predominantly composed of *M.minor*, *T.ophiticola* and *M.orbiculata* respectively, confirming the main pattern found with K = 3, with the difference that a higher proportion of *M.minor* and *T.ophiticola*, but also a majority of *M.oblongifolia* were not assigned to unique clusters. When K = 4 seven and three individuals of *M.minor* and *M.oblongifolia*, respectively, showed probabilities values under 0.9. As for structure, the analysis without considering *M.oblongifolia* with K = 3 displayed very similar results to the analysis including this taxon (Suppl. material 6: fig. S6C); in this case, only one individual of *T.ophiticola* showed probabilities values under 0.9.

### ﻿Integrating morphological and genetic data

**Figure 9.**
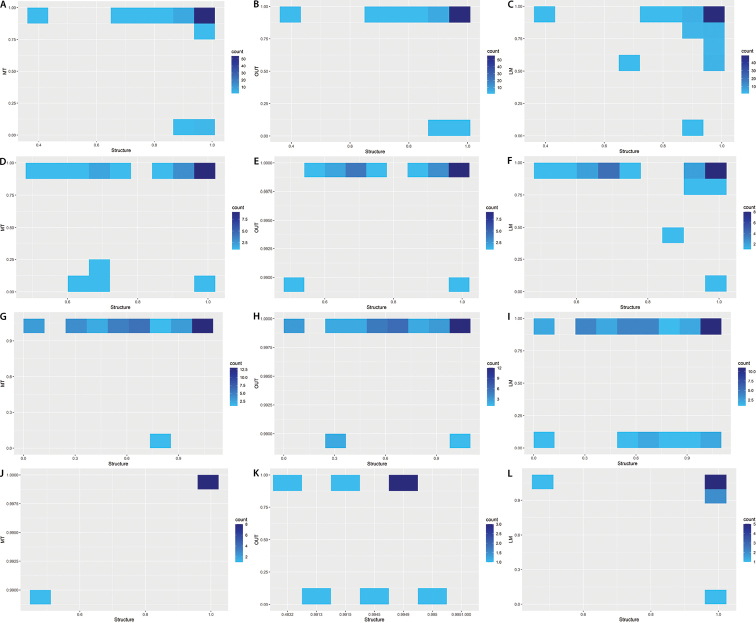
Heatmap with the congruence between morphological (MT: Multivariate, OUT: Elliptic Fourier Descriptors, LM: Matrix of Landmark) and genetic (Structure) cluster probabilities, inside each taxon of Magnoliasubsect.Talauma in Cuba (**A–C**) *Magnoliaminor* (**D–F**) *M.oblongifolia* (**G–I**) *Talaumaophiticola* (**J–L**) *M.orbiculata*. The blue color represents the number of individuals (less individuals: light blue; more individuals: dark blue).

Overall, the morphological and genetic classifications were highly congruent (χ^2^ = 173.69, p < 0.0001). The concordance between the two classifications (genetic and morphology) was especially high for *Magnoliaorbiculata* and *M.minor*, and to a lesser extent for *M.oblongifolia* and *Talaumaophiticola* (Fig. 9). In this last taxon, the classification of several individuals based on genetic markers on one side and leaf traits on the other side were not congruent. Only a few genetic and morphological inconsistencies were also observed in *M.minor* and *M.oblongifolia*.

## ﻿Discussion

### ﻿Morphological variability

The observed leaf morphological variability for Cuban magnolias was higher than that described by previous studies. According to the two taxa CS, the values of leaf length and width were higher than those reported by León and Alain (1950, 1951) for *Magnoliaminor* and *M.orbiculata*. Likewise, in the four taxa CS, these values were higher than what was previously reported by Bisse (1974, 1988), except for *M.oblongifolia*. This difference with previous studies is due to the larger sample size used in the present work and its wider geographic representativeness. In the three taxa CS, Palmarola et al. (2016) reported similar values of length and width for *M.minor* and lower values for *M.oblongifolia* and *M.orbiculata*. The average values of Bisse Index (B_CI_) were similar to those reported by Bisse (1974, 1988) for *M.orbiculata* and *M.oblongifolia*. For *M.minor* and *T.ophiticola*, the average values of B_CI_ are slightly lower and slightly higher, respectively, than those reported by Bisse (1974, 1988). The high level of morphological differentiation between taxa observed in this study reinforces the value of leaf characteristics in taxonomic studies of Cuban magnolias (León and Alain 1950, 1951; Alain 1969; Bisse 1974, 1988; Imkhanitzkaja 1991, 1993; Hernández-Rodríguez 2014; Palmarola et al. 2016). Leaf morphological data are key traits for species delimitation (Jensen et al. 2002; Jensen 2003). This study confirmed they are highly relevant in groups like *Magnolia*, where very little variation is observed in flower and fruit characters (Treseder 1978).

### ﻿An integrative classification of Magnoliasubsect.Talauma in Cuba

In our study, *Magnoliaorbiculata* was clearly distinguished from the other taxa of Magnoliasubsect.Talauma in Cuba based both on morphology and genetic markers. The previously observed large variation of leaf morphology across subsection Talauma in Cuba, although based on the observation of only a few specimens, has been the basis for several authors to consider a unique species in this subsection, therefore including *M.orbiculata* within *M.minor* (Howard 1948; Alain 1969; Borhidi and Muñiz 1971; Lozano-Contreras 1994). In contrast, the present study, as well as lines of evidence already brought by molecular phylogeny of the subsection Talauma (Veltjen et al. 2022) and by studies on the ecological niches of Cuban *Talauma* (Testé et al. in press), strongly supported that *Magnoliaorbiculata* should be considered as a well-delineated species.

However, in our study, a few cases of confusion with *M.minor* (sensu Bisse 1988 and Palmarola et al. 2016) on the basis of leaf morphology traits were observed. This confusion may be explained by the similar rounded shape and relation width-length present in both taxa. Different specialists have erroneously identified some herbarium specimens of *M.orbiculata* as *M.minor* in the past (personal observation in herbarium records). Moreover, our data showed that very few *M.orbiculata* individuals displayed genetic admixture with *M.minor.* Similarly, a few *M.oblongifolia* and *T.ophiticola* individuals displayed genome admixture with *M.orbiculata.* The levels of genetic differentiation among species are influenced by the time of separation and the amount of gene exchange (Hey and Pinho 2012). Genetic variation shared between closely related species may be due to the retention of ancestral polymorphisms because of incomplete lineage sorting (ILS) and/or introgression following secondary contact (Zhou et al. 2017).

Distinguishing between those two causes from observed patterns is challenging, although coalescence modeling can help (e.g. Zhou et al. 2017; Meleshko et al. 2021). However, in the case of *M.orbiculata* relative to other taxa, regular gene flow seems to be unlikely. The very clear morphological and genetic differentiation of *M.orbiculata* with other taxa in Cuba strongly suggested that the lowland between the Sierra Maestra (habitat of *Magnoliaorbiculata*) and Nipe-Sagua-Baracoa (habitat of the other species) may have acted and still acts as a barrier to gene flow by strongly limiting pollination and seed dispersal. Hernández-Rodríguez (2022) reported high levels of genetic differentiation between MagnoliacubensisUrb.subsp.cubensis (from the Sierra Maestra) and *Magnoliacristalensis* Bisse (from Nipe-Sagua-Baracoa), both from subsection Cubenses. Vázquez-García et al. (2016) stated that allopatric speciation seems to be a major driver of *Magnolia* diversification in the Neotropics. Therefore, it seems more likely that the admixture signal between *M.orbiculata* and the other taxa could rather be explained by shared ancestral polymorphism with other Cuban talaumas due to the likely recent diversification of the subsection in Cuba, that is less than 5 mya according to Veltjen et al. (2022), and the recent separation of *M.orbiculata* from the other taxa. However, the possibility of rare events of inter-taxa hybridization involving *M.orbiculata* as one parent cannot be totally ruled out, especially because individuals displaying admixed genome involving *M.orbiculata* have intermediate ancestry coefficients, which is compatible with a hypothetical first- or early-generation hybrid status. Testé et al. (in press) have also shown that the ecological niche of *M.orbiculata* is differentiated from that of the other taxa considered in this study. This may suggest that selection against first- or early-generation hybrids due to local adaptation could also contribute to preventing genetic exchanges between that taxon and the other taxa of Magnoliasubsect.Talauma in Cuba.

Undoubtedly, our data confirmed that the main taxonomic issues concern the north-eastern Cuban populations of Magnoliasubsect.Talauma. León and Alain (1950) have stated that individuals of *M.minor* with more oblong leaves, considered by them as Talaumaminorvar.oblongifolia, may belong to a different species. However, the authors did not assign the species rank to this group because of the absence of reproductive structures in the available specimens. On the other hand, Bisse (1974, 1988) proposed to divide *Magnoliaminor* (sensu León and Alain 1950, 1951) into three separate species (*M.minor*, *M.oblongifolia* and *T.ophiticola*). Our morphological and genetic data did not support those two proposals. Indeed, concerning *M.oblongifolia* (sensu Bisse 1988), the foliar phenotype observed in this taxon appears to be intermediate between *M.minor* and *T.ophiticola*. A recent diversification process or natural hybridization might explain the intermediate characteristics of *M.oblongifolia*, as has been observed for *Quercus* species (Burgarella et al. 2009; An et al. 2017) and the genus *Rhizophora* (Francisco et al. 2018). Rather, considering *M.minor* and *M.oblongifolia* as separate taxa is supported neither by morphological (see Figs 4–7) data nor by genetic data (Fig. 8) of the present study. On the other hand, the existence of a single species, including those three taxa, (*Magnoliaminor* sensu León and Alain 1951) was supported neither by our morphological results, nor by genetic markers, which both showed a clear differentiation between *M.minor* and *T.ophiticola*. Yet, our results did not support either the combination of *Talaumaophiticola* and *Magnoliaoblongifolia* (sensu Bisse 1974, 1988) in a unique taxon, as recently proposed by Palmarola et al. (2016) on the basis of the specimen HFC 5358 from the coast of Moa, which shows both oblong and elliptical leaves. Nevertheless, the delimitation of *T.ophiticola* is still challenging. In the present study, a significant proportion of individuals that were assigned to this taxon based on leaf morphology was unambiguously assigned to the “minor-oblongifolia” genetic cluster, while only one individual of *M.minor* was assigned to the “*ophiticola*” genetic cluster. Also, for each taxon, a significant proportion displayed high genetic admixture between the two genetic clusters identified (and as discussed above rare cases of admixture with *M.orbiculata*). This could be explained by a recent diversification of the three taxa that led to numerous genetic loci with incomplete lineage sorting and to overlaps in the distribution of morphological traits. In trees, factors such as long generation time, and large effective population sizes, increase the opportunity of sharing ancestral polymorphisms through incomplete lineage sorting which makes species identification based on neutral markers even more problematic (Zhou et al. 2017).

The taxa from the north-eastern part of Cuba live in the same habitats and in similar ecological conditions (Testé et al. in press), a situation that is not favorable for the emergence of reproductive barriers. Moreover, those three taxa are also found in sympatry in several locations. The phylogenetic closeness between those three taxa has recently been reported by Veltjen et al. (2022). Therefore, the high admixture level observed in these taxa with SSR markers, as well as the few cases of reciprocal “miss-assignment“, suggest gene flow between the taxa of northeastern Cuba has occurred recently and may still be occurring, producing recombinant and therefore intermediate genotypes and phenotypes. This hypothesis is reinforced by the observation that reciprocal genetic admixture between the two genetic clusters, corresponding mainly to *M.minor* and *T.ophiticola*, is more frequent in the localities where both taxa occur. According to Callaway (1994), hybridization is a common process in magnolias and is more common when the distribution ranges of two or more highly related taxa overlap (Soltis and Soltis 2009).

## ﻿Conclusions

The Cuban taxa of Magnoliasubsect.Talauma showed a high intra-specific leaf morphological variability, which reinforces the value of leaf characteristics in taxonomic studies of Cuban magnolias. As it has been shown in other groups of plants, the integrative approach was efficient to build an accurate classification in Magnoliasubsect.Talauma. Indeed, according to this study, *Magnoliaorbiculata* appears to be an evolutionary lineage separated from other Cuban magnolias of the subsection, with very clear genetic, morphological delimitations, which is consistent with its ecological delimitation already shown (Testé et al. in press). This taxon can thus be considered a true species. Concerning the group of northeastern Cuba taxa, the data supported the existence of two clear groups: corresponding mainly to *M.minor-M.oblongifolia* on the one hand and *T.ophiticola* (sensu Bisse 1988) on the other hand. However, the integrative approach also showed that these two groups cannot be considered as fully delimitated lineages since hybridization between them seems to have occurred recently, or is still ongoing. Because of the likely absence of, at least strong, reproductive barriers between these taxa, we propose therefore to consider them as a species complex.

## Supplementary materials

Supplementary material 1 Graphic representation of the leaf´s morphological variables measured in the individuals of Magnoliasubsect.Talauma in Cuba following the two taxa CS Data type morphological Explanation note The P-values were less than 0.001 in all cases. https://binary.pensoft.net/file/767292This dataset is made available under the Open Database License (http://opendatacommons.org/licenses/odbl/1.0/). The Open Database License (ODbL) is a license agreement intended to allow users to freely share, modify, and use this Dataset while maintaining this same freedom for others, provided that the original source and author(s) are credited.Ernesto Testé

Supplementary material 2 Graphic representation of the leaf´s morphological variables measured in the individuals of Magnoliasubsect.Talauma in Cuba following the three taxa CS Data type morphological Explanation note The P-values were less than 0.001 in all cases; except for the area for the comparison between *M.minor-M.oblongifolia* (p = 0.115) https://binary.pensoft.net/file/767293This dataset is made available under the Open Database License (http://opendatacommons.org/licenses/odbl/1.0/). The Open Database License (ODbL) is a license agreement intended to allow users to freely share, modify, and use this Dataset while maintaining this same freedom for others, provided that the original source and author(s) are credited.Ernesto Testé

Supplementary material 3 Graphic representation of the leaf´s morphological variables measured in the individuals of Magnoliasubsect.Talauma in Cuba following the four taxa CS Data type morphological Explanation note The P-values were less than 0.001 in all cases; except for the perimeter for the comparison between *Magnoliaminor*-*Talaumaophiticola* (p = 0.211) and for the comparison between *M.oblongifolia*-*T.ophiticola* (p = 0.132). https://binary.pensoft.net/file/767294This dataset is made available under the Open Database License (http://opendatacommons.org/licenses/odbl/1.0/). The Open Database License (ODbL) is a license agreement intended to allow users to freely share, modify, and use this Dataset while maintaining this same freedom for others, provided that the original source and author(s) are credited.Ernesto Testé

Supplementary material 4 Graphic representation of the Sum of EDMA and Centroid Size calculated in the individuals of Magnoliasubsect.Talauma in Cuba following the different CS Data type morphological Explanation note The pair *M.minor*-*M.oblongifolia* (p = 0.316) and *M.orbiculata*-*T.ophiticola* (p = 0.406), of the four taxa CS, were the only comparisons with MonteCarlo probabilities over 0.001. https://binary.pensoft.net/file/767295This dataset is made available under the Open Database License (http://opendatacommons.org/licenses/odbl/1.0/). The Open Database License (ODbL) is a license agreement intended to allow users to freely share, modify, and use this Dataset while maintaining this same freedom for others, provided that the original source and author(s) are credited.Ernesto Testé

Supplementary material 5 Structure results of Magnoliasubsect.Talauma in Cuba without *M.oblongifolia* Data type Genetic Explanation note Delta K plot (**A**); The mean Ln(K) plot (**B**); Representative bar plot (out of 100 en replicates) for K = 3 (**C**). https://binary.pensoft.net/file/767296This dataset is made available under the Open Database License (http://opendatacommons.org/licenses/odbl/1.0/). The Open Database License (ODbL) is a license agreement intended to allow users to freely share, modify, and use this Dataset while maintaining this same freedom for others, provided that the original source and author(s) are credited.Ernesto Testé

Supplementary material 6 Discriminant Analysis of Principal Components (DAPC) of Magnoliasubsect.Talauma in Cuba Data type Genetic Explanation note The axes represent the first two linear discriminants. The upper left graph (principal component analysis (PCA) eigenvalues) inset displays the variance explained by the principal component axes used for DAPC and the bottom‐right inset (DA eigenvalues) displays in relative magnitude the variance explained by the two discriminant axes plotted. DAPC graph of the all the taxa (**A–B**), and without *M.oblongifolia* (**C**), 200 principal components (PCs) retained. The individuals with probabilities less than 0.9 were not considered in the contingency tables. https://binary.pensoft.net/file/767297This dataset is made available under the Open Database License (http://opendatacommons.org/licenses/odbl/1.0/). The Open Database License (ODbL) is a license agreement intended to allow users to freely share, modify, and use this Dataset while maintaining this same freedom for others, provided that the original source and author(s) are credited.Ernesto Testé

Supplementary material 7 Historical classification of the Cuban taxa of Magnoliasubsect.Talauma Data type Taxonomy https://binary.pensoft.net/file/767298This dataset is made available under the Open Database License (http://opendatacommons.org/licenses/odbl/1.0/). The Open Database License (ODbL) is a license agreement intended to allow users to freely share, modify, and use this Dataset while maintaining this same freedom for others, provided that the original source and author(s) are credited.Ernesto Testé

Supplementary material 8 Multiplex designed with the 21 microsatellites marker used for the genetic characterization of Magnoliasubsect.Talauma in Cuba Data type Genetic Explanation note Conc.: Concentration; Tm: Primer Melting Temperature (temperature at which one-half of the DNA duplex will dissociate to become single stranded and indicates the duplex stability); Unp: unpublished. https://binary.pensoft.net/file/767299This dataset is made available under the Open Database License (http://opendatacommons.org/licenses/odbl/1.0/). The Open Database License (ODbL) is a license agreement intended to allow users to freely share, modify, and use this Dataset while maintaining this same freedom for others, provided that the original source and author(s) are credited.Ernesto Testé

Supplementary material 9 The Eigenvalue and percent of the total variance of the three first principal components (PC); and the relative weight of each variable to the Principal Component Analysis for the Cuban taxa of Magnoliasubsect.Talauma Data type Morphology Explanation note * The variable with the highest weight per component. https://binary.pensoft.net/file/767300This dataset is made available under the Open Database License (http://opendatacommons.org/licenses/odbl/1.0/). The Open Database License (ODbL) is a license agreement intended to allow users to freely share, modify, and use this Dataset while maintaining this same freedom for others, provided that the original source and author(s) are credited.Ernesto Testé

Supplementary material 10 F-values (F) of the NPMANOVA (one-way) based on Euclidian distance, 10 000 random permutations and Bonferroni-corrected p values (p); calculated on the individuals of Magnoliasubsect.Talauma in Cuba Data type Morphology https://binary.pensoft.net/file/767301This dataset is made available under the Open Database License (http://opendatacommons.org/licenses/odbl/1.0/). The Open Database License (ODbL) is a license agreement intended to allow users to freely share, modify, and use this Dataset while maintaining this same freedom for others, provided that the original source and author(s) are credited.Ernesto Testé

Supplementary material 11 Number of most probable (highest BIC score) groups resulting for the morphological data of the taxa of Magnoliasubsect.Talauma in Cuba Data type Morphology Explanation note Abbreviations: VEV (ellipsoidal, equal shape), EEI (diagonal, equal volume and shape), VVV (ellipsoidal, varying volume, shape, and orientation), EEE (ellipsoidal, equal volume, shape and orientation), BIC (Bayesian information criterion), ICL (integrated complete-data likelihood criterion) https://binary.pensoft.net/file/767302This dataset is made available under the Open Database License (http://opendatacommons.org/licenses/odbl/1.0/). The Open Database License (ODbL) is a license agreement intended to allow users to freely share, modify, and use this Dataset while maintaining this same freedom for others, provided that the original source and author(s) are credited.Ernesto Testé
