# Supplementary figures and images for: ﻿Integrating morphological and genetic limits in the taxonomic delimitation of the Cuban taxa of Magnoliasubsect.Talauma (Magnoliaceae)
Source: PhytoKeys. 2022 Nov 9;213:35–66. doi: 10.3897/phytokeys.213.82627 (PMC9836609; doi:10.3897/phytokeys.213.82627)

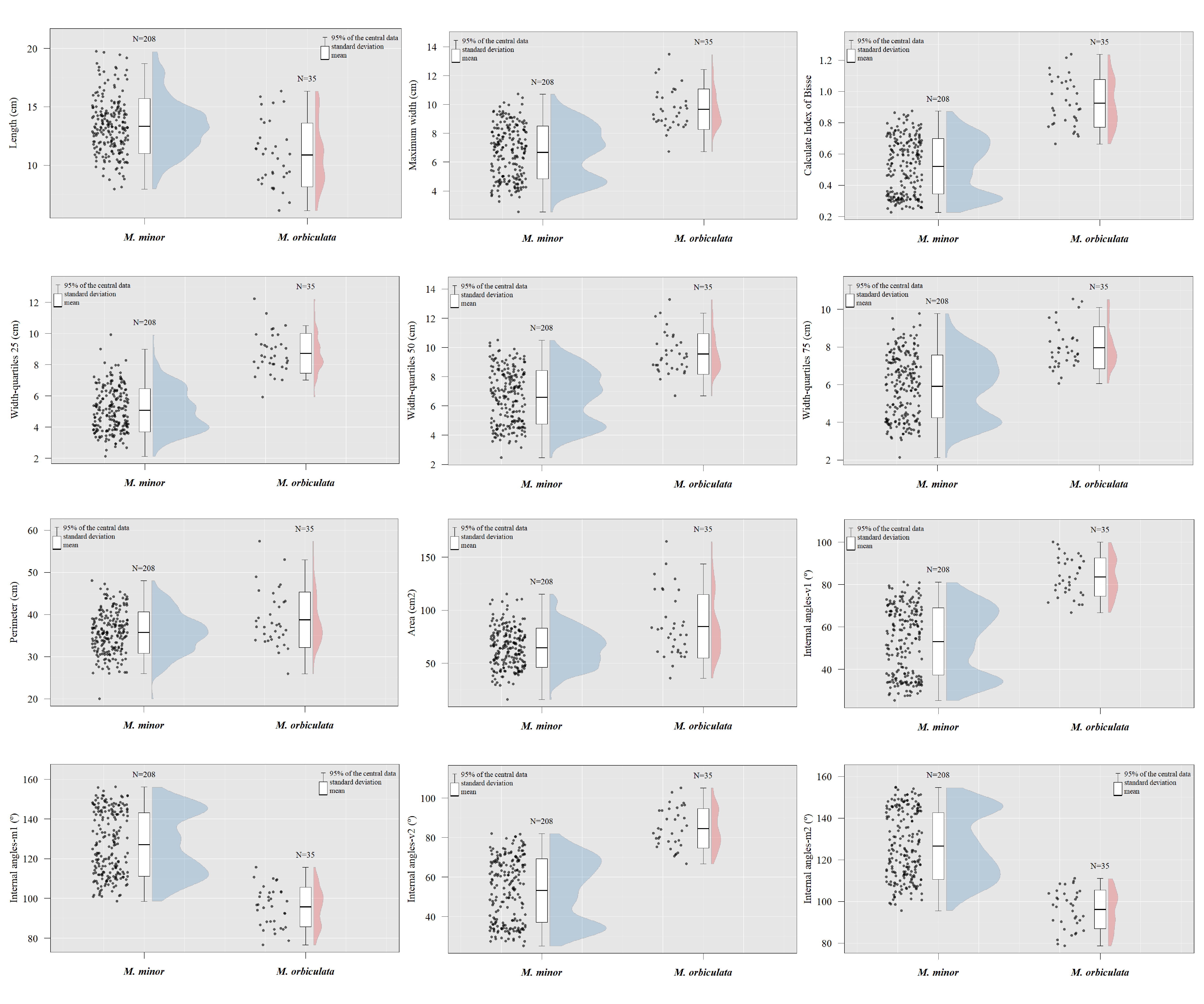

Supplement: Supplementary material 1 — Graphic representation of the leaf´s morphological variables measured in the individuals of Magnoliasubsect.Talauma in Cuba following the two taxa CS [file phytokeys-213-035_article-82627__-s001.jpg]

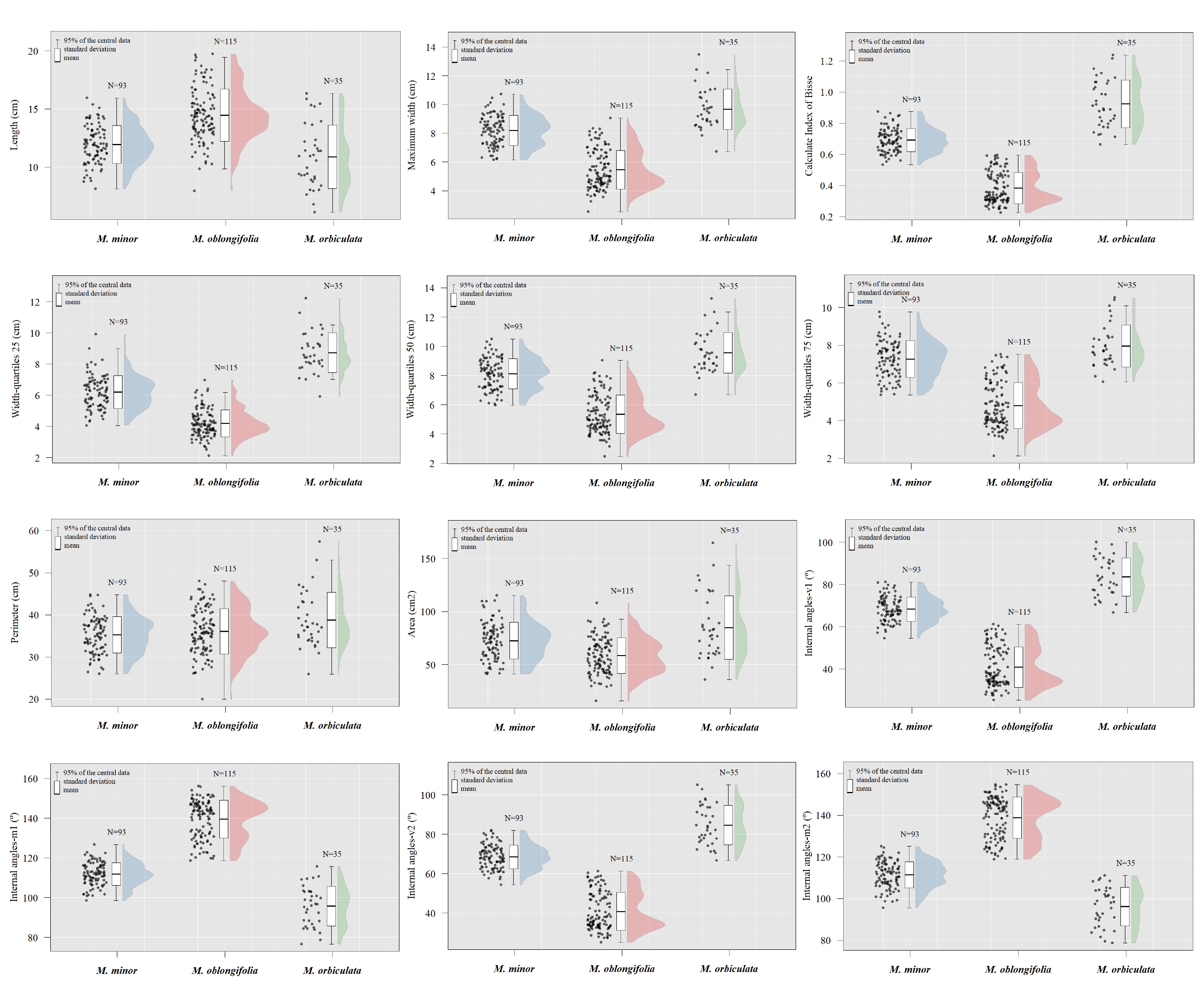

Supplement: Supplementary material 2 — Graphic representation of the leaf´s morphological variables measured in the individuals of Magnoliasubsect.Talauma in Cuba following the three taxa CS [file phytokeys-213-035_article-82627__-s002.jpg]

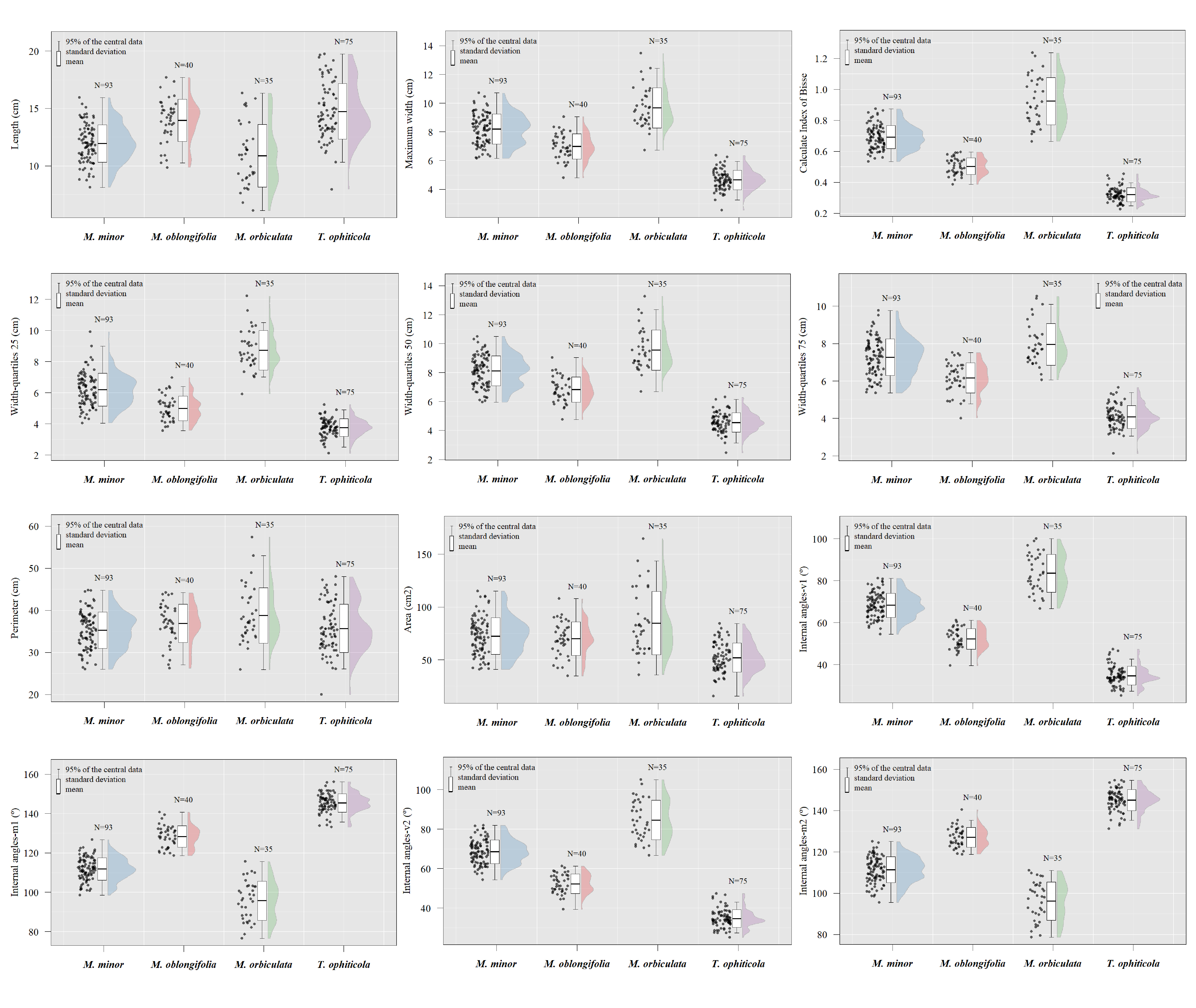

Supplement: Supplementary material 3 — Graphic representation of the leaf´s morphological variables measured in the individuals of Magnoliasubsect.Talauma in Cuba following the four taxa CS [file phytokeys-213-035_article-82627__-s003.jpg]

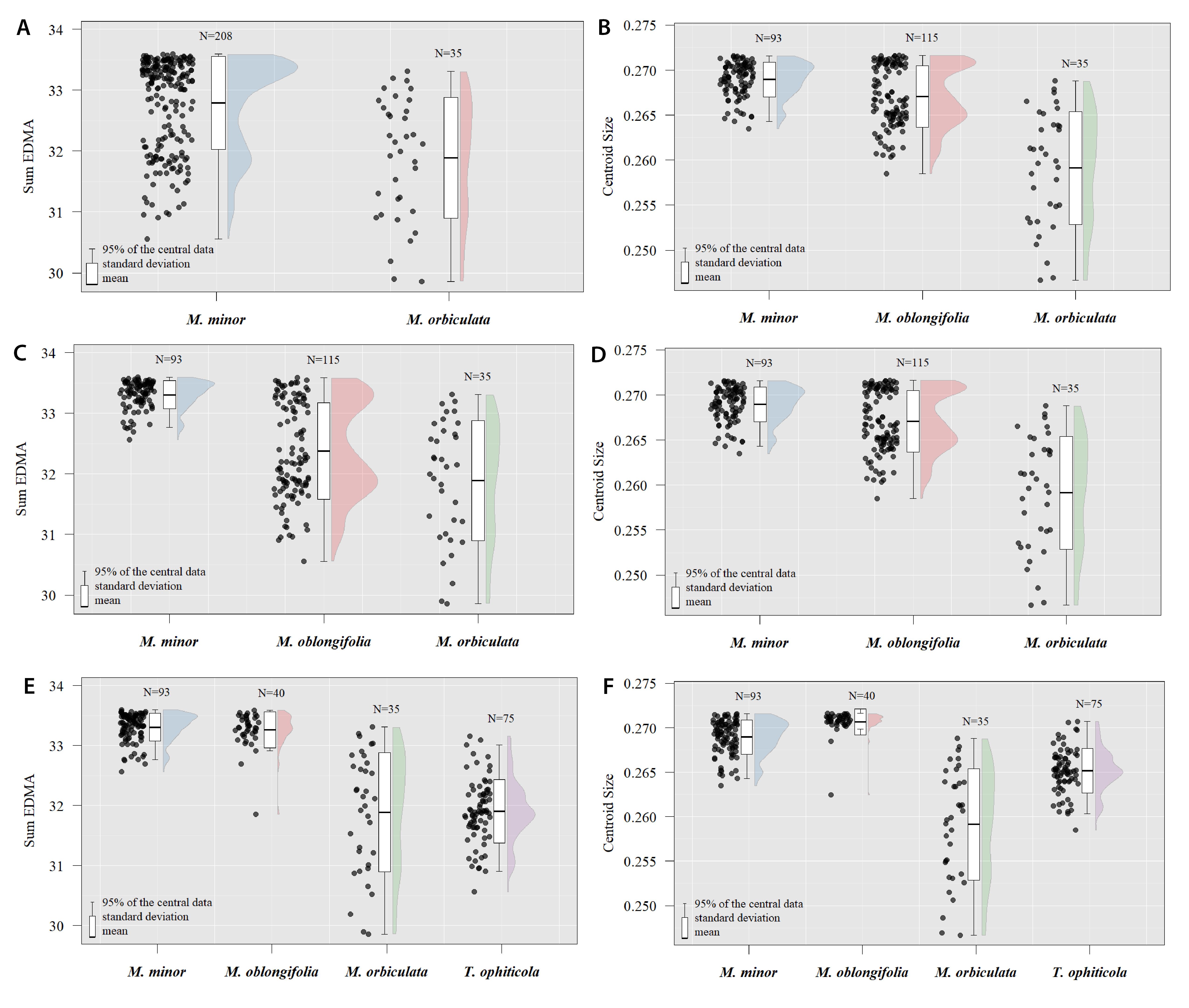

Supplement: Supplementary material 4 — Graphic representation of the Sum of EDMA and Centroid Size calculated in the individuals of Magnoliasubsect.Talauma in Cuba following the different CS [file phytokeys-213-035_article-82627__-s004.jpg]

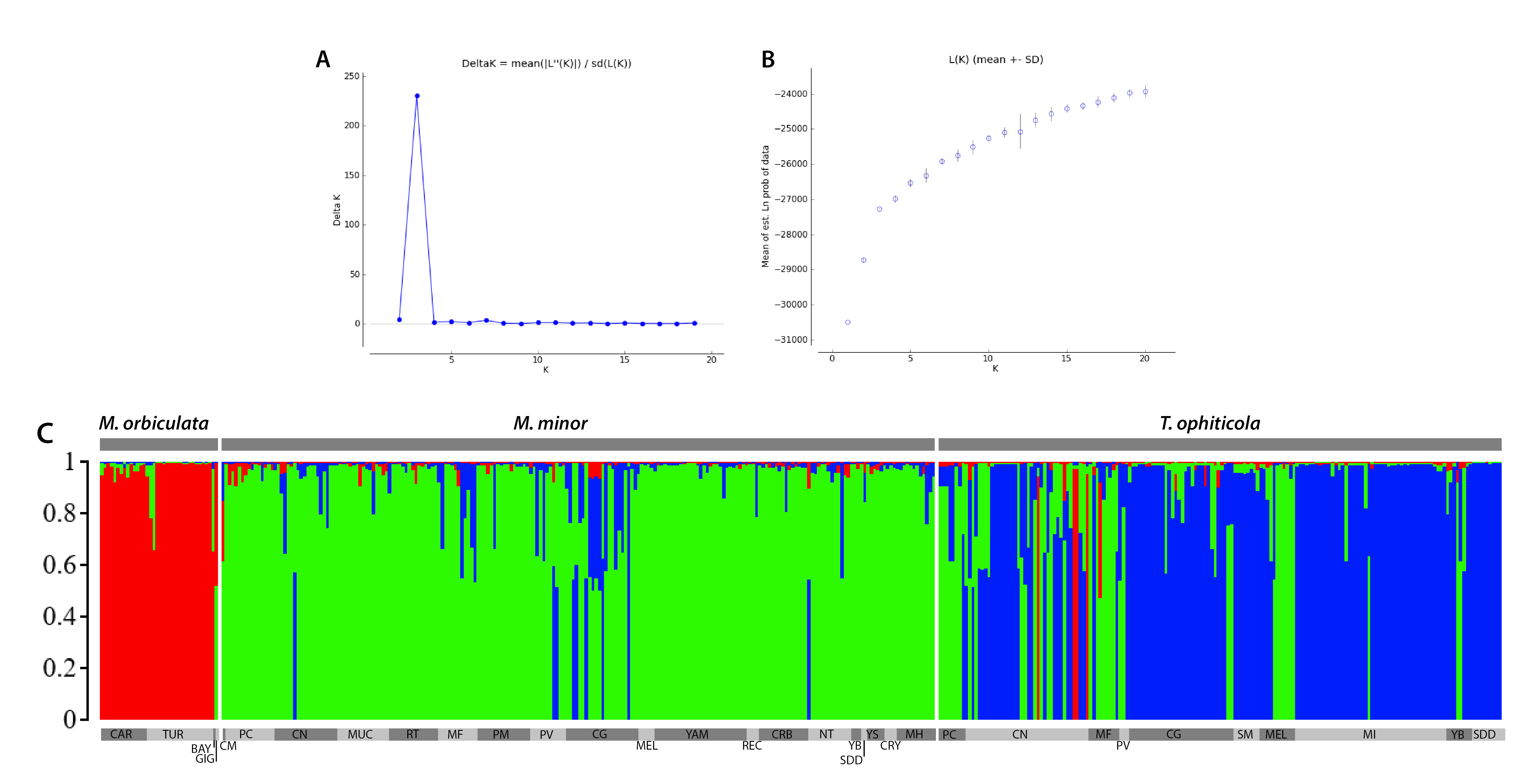

Supplement: Supplementary material 5 — Structure results of Magnoliasubsect.Talauma in Cuba without M.oblongifolia [file phytokeys-213-035_article-82627__-s005.jpg]

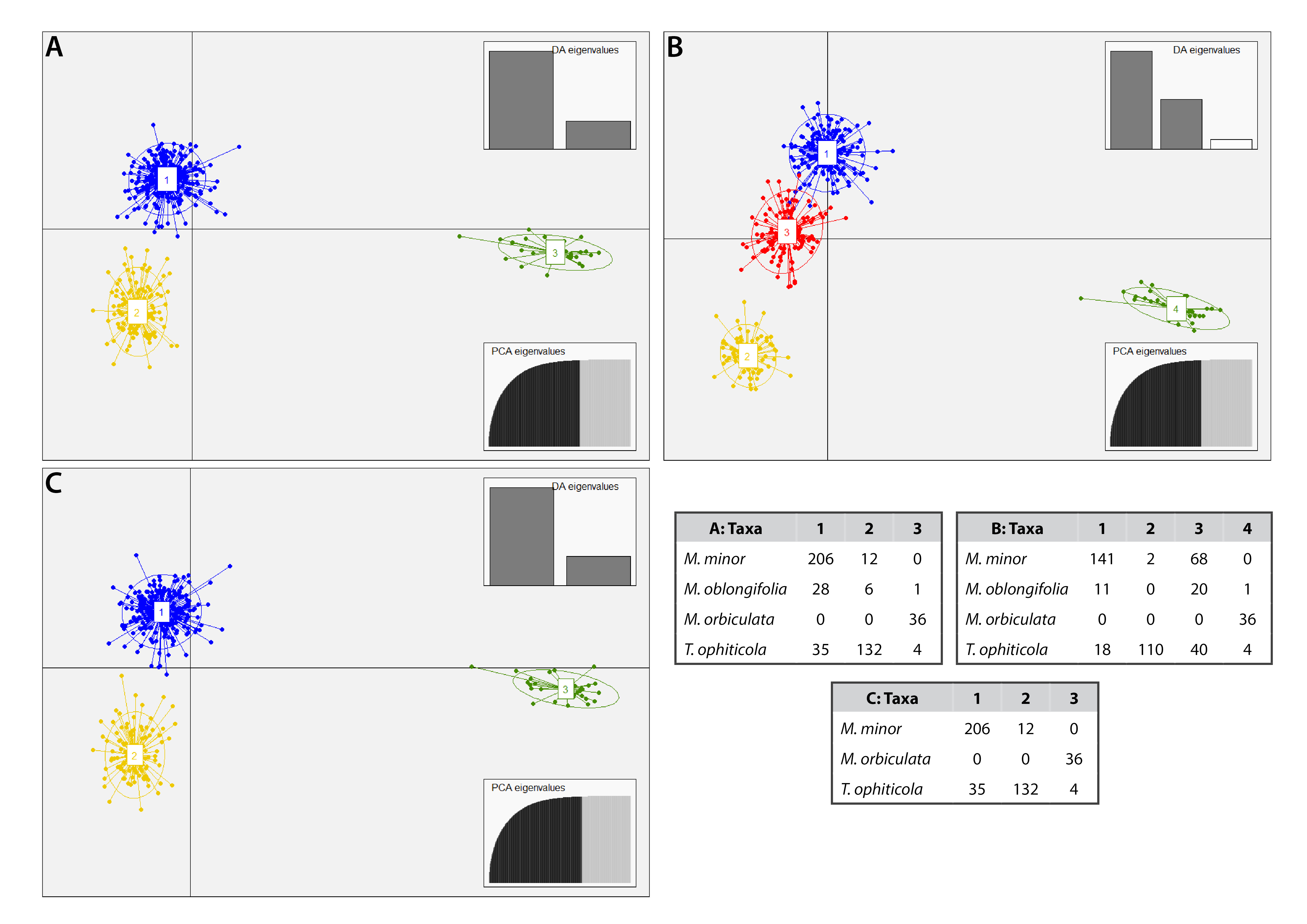

Supplement: Supplementary material 6 — Discriminant Analysis of Principal Components (DAPC) of Magnoliasubsect.Talauma in Cuba [file phytokeys-213-035_article-82627__-s006.jpg]
