# Supplementary material for: ﻿Integrating morphological and genetic limits in the taxonomic delimitation of the Cuban taxa of Magnoliasubsect.Talauma (Magnoliaceae)
Source: PhytoKeys. 2022 Nov 9;213:35–66. doi: 10.3897/phytokeys.213.82627 (PMC9836609; doi:10.3897/phytokeys.213.82627)
Supplement: Supplementary material 7 — Historical classification of the Cuban taxa of Magnoliasubsect.Talauma [file phytokeys-213-035_article-82627__-s007.pdf]

Supplementary Table 1. Historical classification of the Cuban taxa of *Magnolia* subsect. *Talauma*.

ID	Urban (1912)	Britton (1923)	Urban (1927)	Moldenke (1946)	Howard (1948)	León and Alain (1950)
**1**	*Talauma minor* Urb.		*Svenhedinia minor* (Urb.) Urb.		*Talauma minor* Urb.	*Talauma minor* Urb. var. *minor*
**2**						*Talauma minor* var. *oblongifolia* León
**3**						
**4**		*Talauma orbiculata* Britton & P. Wilson				*Talauma orbiculata* Britton & P. Wilson
**5**				*Svenhedinia truncata* Moldenke	*Talauma truncata* (Moldenke) R.A. Howard	

ID	León and Alain (1951)	Alain (1969)	Borhidi and Muñiz (1971)	Bisse (1974, 1988)	Imkhanitzkaja (1993)	Palmarola et al. (2016)
**1**	*Talauma minor* Urb. var. *minor*	*Talauma minor* Urb.	*Talauma minor* Urb. subsp. *minor*	*Talauma minor* Urb.	*Talauma minor* Urb. subsp. *minor*	*Magnolia minor* (Urb.) Govaerts
**2**	*Talauma minor* var. *oblongifolia* León		*Talauma minor* subsp. *oblongifolia* (León) Borhidi	*Talauma* oblongifolia (León) Bisse	*Talauma minor* subsp. oblongifolia (León) Borhidi	*Magnolia oblongifolia* (León) Palmarola
**3**				*Talauma ophiticola* Bisse		
**4**	*Talauma orbiculata* Britton & P. Wilson		*Talauma minor* subsp. *orbiculata* (Britton & P. Wilson) Borhidi	*Talauma orbiculata* Britton & P. Wilson	*Talauma orbiculata* Britton & P. Wilson	*Magnolia orbiculata* (Britton & P. Wilson) Palmarola
**5**	*Talauma truncata* (Moldenke) R.A. Howard					
