# Supplementary material for: ﻿Integrating morphological and genetic limits in the taxonomic delimitation of the Cuban taxa of Magnoliasubsect.Talauma (Magnoliaceae)
Source: PhytoKeys. 2022 Nov 9;213:35–66. doi: 10.3897/phytokeys.213.82627 (PMC9836609; doi:10.3897/phytokeys.213.82627)
Supplement: Supplementary material 8 — Multiplex designed with the 21 microsatellites marker used for the genetic characterization of Magnoliasubsect.Talauma in Cuba [file phytokeys-213-035_article-82627__-s008.pdf]

Supplementary Table 2. Multiplex designed with the 21 microsatellites marker used for the genetic characterization of *Magnolia* subsect. *Talauma* in Cuba. Conc.: Concentration; Tm: Primer Melting Temperature (temperature at which one-half of the DNA duplex will dissociate to become single stranded and indicates the duplex stability); Unp: unpublished.

Marker (Multiplex)	Conc. (uL)	Forward primer<br>Reverse primer	Tm	Amplicon size	Motif (No. repeats)	GenBank Accession Number
MA39_120_M13 (1)	1	F: GTGAGAACCGGTGGACAGTT<br>R: ATGCATGTACAAAGGTGCGC	F: 59.894<br>R: 59.829	96	AC (15)	Unpublished
MA41_120_M13 (1)	1	F: ATTGTCACAGGTGTGGAGGC<br>R: AAGTCTGGCTCCCAATGGTG	F: 60.251<br>R: 59.961	143	AG (17)	Unpublished
MA42_421_M13 (1)	3	F: GACAGCAGACCTGACCGATT<br>R: GACCAGTGCATCCCATCAAA	F: 59.752<br>R: 58.162	280	AG (10)	MH923428
MA42_072_Hill (1)	2	F: CCCACCTAGGTTTCCAGTGC<br>R: TGCGTTCGAAAGGCACAATG	F: 60.323<br>R: 60.041	245	AC (5)	MH923401
MA39_280_Neo (1)	2	F: AGCCGAACAGTCACTCCATC<br>R: AGTGGCATTTCACCGCAATG	F: 59.752<br>R: 59.756	144	AG (14)	Unpublished
MA42_495_Neo (1)	2	F: TGCATCTCCTCATCCTCCCA<br>R: ACGCCATTCAATTACCTACGG	F: 60.031<br>R: 58.443	97	AG (26)	MH923433
MA39_182_T3 (1)	1	F: CTACACGGGTGAAGCCTACC<br>R: GGCCGTAATCAGAGTCCACC	F: 59.825<br>R: 60.179	129	AG (12)	MH923376
MA40_045_M13 (2)	1	F: TTGTGGGCCAAGCTCGATAG<br>R: ATTGTGGCATGTACCTCGCA	F: 60.108<br>R: 60.036	232	AG (13)	MH923387
MA42_130_Hill (2)	1	F: CAGTCGACCGACCTCTCAAC<br>R: CACGTCTGATGCCTCCTCAC	F: 60.11<br>R: 60.459	96	AG (22)	Unpublished
MA42_150_Neo (2)	2	F: TGCTCAGTGCTCACATGAGG<br>R: CCGGTGTTCACCTGAGCATT	F: 60.037<br>R: 60.606	94	AC (15)	Unpublished
MA39_259_T3 (2)	1	F: TGATAGAGTGGGATGGCGGA<br>R: TTGGTTCATGCATCGGTCCT	F: 60.106<br>R: 59.672	96	AG (11)	MH923380
MA42_255_M13 (3)	1	F: ACGTGGGTCGAGGATCAAGT<br>R: GGACCCACCTCCAACAGATC	F: 60.899<br>R: 59.747	137	AG (14)	MH923417
MA42_231_Hill (3)	0.5	F: GGGTGCGAAATGTGCATCAA<br>R: GGGCCAGTGAGCATTAGAGC	F: 59.757<br>R: 60.817	131	AG (14)	MH923413
MA42_028_Neo (3)	1	F: GGATCGTCTTCCGCCATTCT<br>R: TTCCGTACGATGCTCCCATG	F: 59.895<br>R: 59.896	151	AG (33)	MH923398

MA39_191_Neo (3)	1	F: TCCAACGAGTACTTGGGCAG<br>R: GATGCGTCCTTGAGTCCCAA	F: 59.68<br>R: 60.036	171	AG (22)	Unpublished
MA42_441_M13 (4)	1	F: TTGACTGCATCTCCCTTCCT<br>R: CTCATCTCCGCTTCAGCAGG	F: 58.339<br>R: 60.53	305	AG (5)	Unpublished
MA42_471_M13 (4)	1	F: TGATGAAGAGCCCAGATCGTC<br>R: TGGCCTTGTTCTCCATACGT	F: 59.589<br>R: 59.019	153	AG (16)	MH923429
MA41_373_Hill (4)	1	F: GCGCCCAATCAGAACACAAC<br>R: GGGAAGAGCTTCTTTCGCCA	F: 60.387<br>R: 60.322	165	AG (16)	MH923396
MA39_442_Neo (4)	1	F: AGTCGATCCTCTTGCTGCAC<br>R: GAGGGAGCATCGGCCATTAC	F: 60.108<br>R: 60.604	109	AAG (8)	MH923386
MA39_185_Neo (4)	1	F: CGGGTGTTGTAGATGACGCT<br>R: AAGACACGGAATGGGACGAG	F: 60.109<br>R: 59.753	209	AG (15)	MH923377
MA39_342_T3 (4)	1	F: TCCCTTCAGTCTTCACACGC<br>R: AAAGGAGCGTTGAGTGGTGG	F: 59.966<br>R: 60.535	146	AG (14)	MH923384
