# Supplementary material for: ﻿Integrating morphological and genetic limits in the taxonomic delimitation of the Cuban taxa of Magnoliasubsect.Talauma (Magnoliaceae)
Source: PhytoKeys. 2022 Nov 9;213:35–66. doi: 10.3897/phytokeys.213.82627 (PMC9836609; doi:10.3897/phytokeys.213.82627)
Supplement: Supplementary material 9 — The Eigenvalue and percent of the total variance of the three first principal components (PC); and the relative weight of each variable to the Principal Component Analysis for the Cuban taxa of Magnoliasubsect.Talauma [file phytokeys-213-035_article-82627__-s009.pdf]

Supplementary Table 3. The Eigenvalue and percent of the total variance of the three first principal components (PC); and the relative weight of each variable to the Principal Component Analysis for the Cuban taxa of *Magnolia* subsect. *Talauma*. * The variable with the highest weight per component.

	Eigenvector		
	PC-1	PC-2	PC-3
Eigenvalue	8.87	2.79	0.18
Variance (%)	73.90	23.28	1.52
Variables			
Length	0.1651	0.5140	-0.0435
Maximum width	-0.3208	0.1607	-0.2513
Calculated Index of Bisse	-0.3200	-0.1605	0.2134
Width-quartile 25	-0.3100	0.1407	0.5557*
Width-quartile 50	-0.3220	0.1549	-0.1740
Width-quartile 75	-0.3145	0.1527	-0.5180
Perimeter	-0.0763	0.5627*	0.4639
Area	-0.2101	0.4570	-0.2318
Internal angles-v1	-0.3245*	-0.1493	0.0386
Internal angles-m1	0.3233	0.1485	-0.0869
Internal angles-v2	-0.3237	-0.1537	0.0622
Internal angles-m2	0.3227	0.1536	-0.0134
