# Supplementary material for: ﻿Integrating morphological and genetic limits in the taxonomic delimitation of the Cuban taxa of Magnoliasubsect.Talauma (Magnoliaceae)
Source: PhytoKeys. 2022 Nov 9;213:35–66. doi: 10.3897/phytokeys.213.82627 (PMC9836609; doi:10.3897/phytokeys.213.82627)
Supplement: Supplementary material 10 — F-values (F) of the NPMANOVA (one-way) based on Euclidian distance, 10 000 random permutations and Bonferroni-corrected p values (p); calculated on the individuals of Magnoliasubsect.Talauma in Cuba [file phytokeys-213-035_article-82627__-s010.pdf]

Supplementary Table 4. F-values (F) of the NPMANOVA (one-way) based on Euclidian distance, 10 000 random permutations and Bonferroni-corrected p values (p); calculated on the individuals of *Magnolia* subsect. *Talauma* in Cuba.

	**F**	**p**
**Linear and angular dataset**		
Two taxa CS	92.28	< 0.001
Three taxa CS	212.5	< 0.001
Four taxa CS	206.6	< 0.001
**Outline dataset**		
Two taxa CS	156.9	< 0.001
Three taxa CS	398.8	< 0.001
Four taxa CS	516	< 0.001
**Landmark dataset**		
Two taxa CS	272.1	< 0.001
Three taxa CS	360.7	< 0.001
Four taxa CS	432	< 0.001
