# Supplementary material for: ﻿Integrating morphological and genetic limits in the taxonomic delimitation of the Cuban taxa of Magnoliasubsect.Talauma (Magnoliaceae)
Source: PhytoKeys. 2022 Nov 9;213:35–66. doi: 10.3897/phytokeys.213.82627 (PMC9836609; doi:10.3897/phytokeys.213.82627)
Supplement: Supplementary material 11 — Number of most probable (highest BIC score) groups resulting for the morphological data of the taxa of Magnoliasubsect.Talauma in Cuba [file phytokeys-213-035_article-82627__-s011.pdf]

Supplementary Table 5. Number of most probable (highest BIC score) groups resulting for the morphological data of the taxa of *Magnolia* subsect. *Talauma* in Cuba. Abbreviations: VEV (ellipsoidal, equal shape), EEI (diagonal, equal volume and shape), VVV (ellipsoidal, varying volume, shape, and orientation), EEE (ellipsoidal, equal volume, shape and orientation), BIC (Bayesian information criterion), ICL (integrated complete-data likelihood criterion)

G	Model	Log-likelihood	BIC	ICL
**Linear and angular dataset**				
G=2	VEV	1834.648	2735.475	2727.775
G=3	VEV	2368	3362.735	3361.879
G=4	VEV	2617.274	3421.837	3418.993
G=5	VEV	2783.715	3315.276	3314.166
G=6	VEV	2840.613	2989.626	2987.493
G=7	VEV	2951.995	2772.946	2771.089
G=8	VEV	3124.385	2678.279	2676.927
G=9	VEV	3250.297	2490.66	2488.738
**Elliptic Fourier Descriptors dataset**				
G=2	EEI	151,956.5	302,259.6	302,259.6
G=3	EEI	152,188.4	302,168.6	302,168.6
G=4	EEI	152,358.6	301,954.1	301,954.1
G=5	EEI	153,380.4	303,443	303,442.9
G=6	EEI	153,610.6	303,348.6	303,346
G=7	EEI	153,649.5	302,871.7	302,871.3
G=8	EEI	153,812.5	302,642.9	302,642.9
G=9	EEI	153,953.5	302,370	302,370
**Matrix of Landmark dataset**				
G=2	VVV	44,329.82	83,886.17	83,886.07
G=3	EEE	43,324.66	83,946.72	83,945.98
G=4	EEE	43,375.32	83,888.76	83,882.96
G=5	EEE	43,410.8	83,800.42	83,781.46
G=6	EEE	44,014.69	84,848.89	84,828.99
G=7	EEE	44,037.63	84,735.48	84,716.24
G=8	EEE	44,101.98	84,704.88	84,688.31
G=9	EEE	44,197.58	84,736.77	84,715.57
